# Partners in Health: Investigating Social Genetic Effects Among Married and Cohabiting Couples

**DOI:** 10.1007/s10519-023-10147-w

**Published:** 2023-06-07

**Authors:** Kasper Otten, Jornt J Mandemakers

**Affiliations:** 1 Department of Sociology, Utrecht University, Padualaan 14, 3584 CH Utrecht, the Netherlands; 2 Atlas Research, Amsterdam, the Netherlands

**Keywords:** Social genetic effects, Social contagion, Genetics, Health, Partners

## Abstract

Partners resemble each other in health behaviors and outcomes such as alcohol use, smoking, physical activity, and obesity. While this is consistent with social contagion theory suggesting partner influence, it is notoriously difficult to establish causality because of assortative mating and contextual confounding. We offer a novel approach to studying social contagion in health in long-term partnerships by combining genetic data of both partners in married/cohabiting couples with longitudinal data on their health behaviors and outcomes. We examine the influence of the partner’s genetic predisposition for three health outcomes and behaviors (BMI, smoking, and drinking) among married/cohabiting couples. We use longitudinal data from the Health and Retirement Study and the English Longitudinal Study of Ageing with data on health outcomes and genotypes for both partners. Results show that changes over time in BMI, smoking, and drinking depend on the partner’s genetic predispositions to these traits. These findings underline the importance of people’s social surroundings for their health and highlight the potential of targeting health interventions at couples.

**Supplementary Information:**

The online version contains supplementary material available at 10.1007/s10519-023-10147-w.

## Introduction

The partner’s lifestyle has considerable associations with one’s own lifestyle across a variety of domains, including obesity, physical activity, eating, alcohol consumption, weight loss, and smoking (Meyler et al. 2007; Richmond-Rakerd and Belsky 2017). Partners share resources and they may exert a positive influence by promoting a healthy lifestyle and by sanctioning unhealthy behavior (Franks et al. 2002; Conklin et al. 2014; Jackson et al. 2015; Margolis and Wright 2016; Umberson et al. 2018), although partners may also reinforce unhealthy behaviors. These findings align with the ‘social contagion’ strand of research, which shows that health behaviors spread through social networks, including dyadic ties such as between partners (Christakis and Fowler 2007, 2008, 2013; Rosenquist et al. 2010). However, it is notoriously difficult even in longitudinal research designs to establish causality because of assortative mating (‘like seeks like’) and contextual confounding (VanderWeele 2011). Although natural experiments have been used to examine social contagion effects (e.g., student dorms; Guo et al. 2015; Li and Guo 2016), they are rare, and we know of no such experiment for partners (fortunately by the way).

In this paper, we offer a novel approach to studying social contagion for health behaviors in long-term partnerships by utilizing longitudinal data on health behaviors and outcomes of both partners in married/cohabiting couples combined with their genetic profiles. Genetics offers a unique angle to examine the partner’s influence because genes are randomly assigned at conception given parental genotypes, are not directly visible, can be measured, and have an ongoing influence on the phenotype. This means that the partner’s genotype, as opposed to his/her phenotype, is immune to influences from the couple environment and reverse causality through influences from ego after partner selection. In selecting partners, people may still have an indirect influence on their partner’s genotype through selecting on similar phenotypes. This is why we use longitudinal data to control for phenotypes earlier in the relationship and assess whether a partner’s genotype influences the change in ego’s phenotype over time. The impacts of genotypes of others in our direct environment on our own phenotypes have been referred to as social genetic effects or indirect genetic effects (Baud et al. 2017; Domingue and Belsky 2017). We study social genetic effects for probably the most important relationship in adulthood; the partner. Despite the importance of the partner for our health and well-being and decades of research, our understanding of its role is still limited (Meyler et al. 2007; Carr and Springer 2010). This study examines the influence of the partners’ genotype for one’s health behaviors and outcomes among married/cohabiting couples.

Although social genetic effects have been established for numerous types of animals (Ellen et al. 2014; Nielsen et al. 2014; Baud et al. 2017), trees (Brotherstone et al. 2011), and bacteria (Lee et al. 2010), and are sometimes found to be even stronger than direct genetic effects (Baud et al. 2017), they were until recently virtually ignored in studies on human genetics (but see these recent studies; Brunello et al. 2020; Das 2019; Harris et al. 2018; Kong et al. 2018; Laidley et al. 2019; Liu 2018; Salvatore et al. 2020; Sotoudeh et al. 2019; Xia et al. 2021). Because married/cohabiting people are exposed to their partner’s behavior (ego’s environment), which is partly driven by the partner’s genes, we may be able to detect social genetic effects. For instance, having a partner who is genetically predisposed to alcohol dependence may increase one’s own alcohol consumption because the partner ensures there is alcohol available at home. What is more, social genetic effects may be particularly likely for partners compared to other social ties because partners face large costs to leaving their relationship and are typically exposed to each other on a daily basis, which may increase incentives to influence the partner’s behavior and also the willingness to conform.

Previous research examined social genetic effects by estimating polygenic indexes (PGI; the aggregation of many small genetic effects scattered across the genome on a phenotype) for both partners and testing whether ego’s phenotype is associated with the PGI of ego’s partner while controlling for ego’s own PGI. However, this approach is known to suffer from confounding by assortative mating (Xia et al. 2021). A PGI is a noisy estimate of the genetic component of a trait, and does not capture the full genetic component. Controlling for ego’s own PGI therefore only controls for a small fraction of ego’s own genetic component. Furthermore, even if one could control for one’s full genetic component, this would not remove the influence of assortative mating because people mate on the basis of phenotypes that are not completely heritable. For these reasons, a gene-environment (GxE) correlation (Abdellaoui et al. 2022) between the PGI of the partner and the phenotype of ego is expected even after controlling for ego’s full genetic component. Such GxE correlations have also been referred to as “social genetic correlations” (Harris et al. 2018) and have to be distinguished from social genetic effects in which the partner’s genotype influences ego’s behavior (in line with the partner social contagion hypothesis). Indeed, a recent study on social genetic effects in humans suggests that the biggest remaining challenge is to account for assortative mating and calls for future research using longitudinal analysis (Xia et al. 2021). We use longitudinal data on phenotypes to control for phenotypes earlier in the relationship and assess whether a partner’s genotype influences the change in ego’s phenotype over time. We believe we are the first to provide estimates for social genetic effects in health behaviors and outcomes while explicitly accounting for assortative mating.

We use data from the Health and Retirement Study (HRS) and the English Longitudinal Study of Ageing (ELSA). Both studies follow a nationally representative sample of adults aged 50 and older every two years. HRS does this for people in the United States since 1992 and ELSA for people in England since 2002. DNA samples have been collected in both studies, and if a participant has a partner, this partner is automatically selected to participate in the study as well, even if he or she is younger than 50 years. We examine three outcomes for which extensive information on genetics is available and that are important indicators of a healthy lifestyle; namely BMI (Yengo et al. 2018b), as a measure of adiposity, and two measures of health behavior: smoking (cigarettes per day, CPD) (Liu et al. 2019), and the level of alcohol consumption (natural log of drinks per week left anchored at 1, DPW) (Liu et al. 2019). We start by examining partner similarity on a phenotypic and genotypic level for these outcomes. Recent findings show weak genetic similarity between partners for education, height, and BMI (Sebro et al. 2010; Domingue et al. 2014; Guo et al. 2014; Abdellaoui et al. 2015; Zoua et al. 2015; Conley et al. 2016; Hugh-jones et al. 2016; Robinson et al. 2017; Yengo et al. 2018a), but to our knowledge no research examined similarity in drinking and smoking behavior across the genome. We then build on the strength of the longitudinal design of the HRS and ELSA to achieve a strict control for assortative mating by conditioning on initial observed levels of health behavior of ego. Because men and women differ in healthy lifestyle and the social and genetic influences on health may be different for men and women (Short et al. 2013), we also include sex-stratified analyses.

## Methods and Materials

### Data

The Health and Retirement Study (HRS) is a longitudinal household study that follows a nationally representative sample of adults aged 50 and older in the United States every two years since 1992 (Sonnega et al. 2014). The English Longitudinal Study of Ageing (ELSA) is a longitudinal household study that follows a nationally representative sample of adults aged 50 and older in England every two years since 2002 (Steptoe et al. 2013). If a participant had a partner, this partner is automatically selected to participate in the study as well. We limited the sample to different-sex married/cohabiting couples of European descent where both partners had valid genomic data and excluded proxy interviews. Only couples of whom both partners were of European descent are included in the analysis because polygenic indexes were based on European GWAS and have reduced predictive power in other ancestries. We further excluded observations of couples in case one or both partners was no longer living independently, but for instance in a nursing home. We used listwise deletion of missing values on the main variables of interest (BMI/drinks per week/smoking, sex, age and educational level for both spouses). The HRS sample comprises years 1992–2018 and the ELSA sample years 2002–2019. For BMI, the analytical sample comprised 59,325 observations from 9522 persons in 5879 couples in the HRS and 11,728 observations from 4311 persons in 2729 couples in the ELSA. For drinks per week, the analytical sample comprised 52,023 observations from 9140 persons in 5584 couples in the HRS and 24,179 observations from 4911 persons in 3264 couples in the ELSA. For smoking, the analytical sample comprised 60,029 observations from 9546 persons in 5885 couples in the HRS and 25,740 observations from 4943 persons in 3311 couples in the ELSA. Note that the sample differs somewhat between outcomes (see Tables S1 and S2 in the supplementary material for details). This study was conducted with institutional review board approval from Utrecht University, the Netherlands.

### Measures

All measurements are made for ego and partner. BMI (Body Mass Index) is measured by dividing weight in kilograms by length in meters squared. Drinking is measured by the number of alcoholic drinks per week (DPW). Smoking was measured by the number of cigarettes per day (CPD) and non-smokers were set to zero. We take the natural logarithm for DPW, left anchored at 1 (ln(y + 1)).

Genetic propensity for BMI/DPW/CPD is measured by the use of polygenic indexes (PGI). A PGI is the aggregation of many small genetic effects scattered across the genome on a phenotype. It is computed by weighting the alleles at the different loci across the genome with their association to the phenotype of interest, and then summing these weighted alleles. Information on the association between alleles and phenotypes is derived from recent large-scale publicly available GWAS (Yengo et al. 2018b; Liu et al. 2019) that did not overlap with the HRS/ELSA.

We used the polygenic index (PGI) for each outcome that was available in the public domain and that was created in an identical way for both HRS and ELSA to facilitate reproducibility and to maximize comparability. The PGIs were available through the Polygenic Index Repository or the HRS and/or ELSA studies (Ajnakina and Steptoe 2019; Banks et al. 2021; Becker et al. 2021; Ware et al. 2021). For BMI and DPW we used the single-trait PGIs from the repository (Becker et al. 2021). For CPD we used the PGI (Ajnakina and Steptoe 2019; Ware et al. 2021) based on the GSCAN GWAS (Genome-Wide Association Study) of smoking behavior (Liu et al. 2019), as it proved to be more predictive than the repository’s PGI in our analytical samples. The derivation of all the polygenic indices in the HRS and ELSA was identical. We used the first 20 principal components (PCs) for the partner to account for population stratification (Price et al. 2006; Becker et al. 2021; Ware et al. 2021). We also include analyses where we additionally control for the first 20 principal components of ego.

### Analyses

We examine social genetic effects by estimating the effect of the partner’s PGI on ego’s phenotype (BMI/CPD/DPW) using random effect regression models controlling for ego’s first observed scores for each phenotype in the partnership (BMI/CPD/DPW respectively). For example, if ego’s phenotype is first observed in wave 2, then this first phenotype will be controlled for in the subsequent waves and ego will only be included in the analyses after wave 2. In addition, the analyses control for PCs of the partner, which provides a broad control for genetic similarity between partners. Individuals can occur as both ego and partner in the data, so each couple can occur twice at each wave (directed dyads). We adjust for repeated observations within individuals/couples over time by estimating a random intercept for dyads (directed). We use robust standard errors clustered on the household level based on sandwich estimators (White 1982) because husband and wife can both be ego and the partner and some individuals have had more than 1 partner in the course of the study. We furthermore control for ego’s sex (which also captures the sex of the partner because we only include different-sex couples), both ego’s and partner’s age (linear and squared), sex-by-age interactions, and year of observation dummies and relationship length to capture secular trends and main demographic differences in health behavior. For each model, we include a version with and without controlling for ego’s own PGI and PCs. All models were also estimated for husbands and wives separately to investigate whether results were mainly driven by husbands influencing wives or vice versa.

We conducted sensitivity analyses to evaluate whether findings are affected by outliers in BMI (< 20 or > 40), heavy smokers (≥ 20 CPD), or heavy drinkers (≥ 28 drinks/week in the ELSA and > 14 in the HRS, which is equivalent to the top ~ 5% in the ELSA/HRS), and by excluding non-drinkers and non-smokers. In addition, we performed analyses in which we control for the baseline difference between partners in the phenotype of interest (BMI, drinking, or smoking). The results of these analyses were not substantially different from the main analyses (see Figure S3 and Table S11 in the supplementary material for details). We also conducted analyses in which we interact the social genetic effects with the number of years between the first and the current ego phenotype. We find that this interaction is insignificant for all outcomes (Table S10 in the supplementary material).

We repeat our social genetic effects analysis but for height as phenotype, as a negative control. We should not expect a social genetic effect for height because height is largely fixed at the time of partner choice, but there is assortative mating on height (Stulp et al. 2017). Hence, if our analyses are successful at ruling out assortative mating – by conditioning on ego’s first observed phenotype – we should obtain a null result for height. The social genetic effect analysis for height thus presents a negative control to assess the validity of the results obtained for the other phenotypes (BMI/CPD/DPW). The results of this negative control analysis are reported in the supplementary material (Figure S4 and Table S7) and indeed show a null result for height.

Our analyses on the association between the partner’s polygenic index and ego’s phenotype do not assume that the effect goes directly and only through the partner’s phenotype. At the same time, the partner’s phenotype is plausibly a main mechanism through which the partner’s polygenic index has an effect. We examine this via instrumental variable (IV) analyses, in which the partner’s polygenic index (for smoking, drinking, and BMI) acts as an instrumental variable for the partner’s actual health behavior or outcome (smoking, drinking, and BMI). We also conduct multilevel multivariate analyses, in which data from the three different phenotypes (smoking, drinking, and BMI) are combined into one analysis per data source (HRS and ELSA). Finally, we conduct analyses using change scores, where the outcome variable is the difference between ego’s current behavior and the behavior at baseline.

## Results

### How Similar are Partners?

**Fig. 1:**
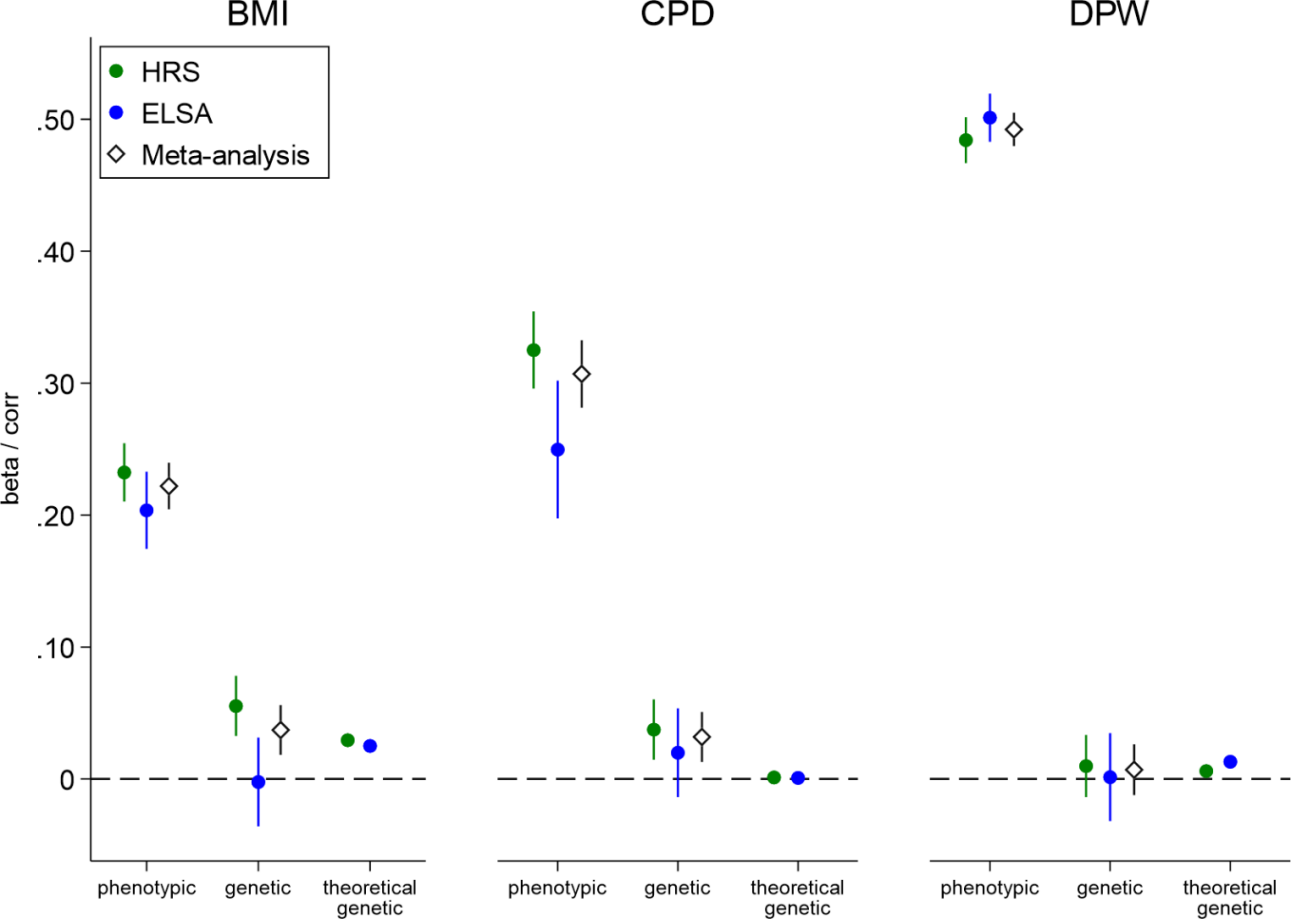
Phenotypic and genetic similarity between partners. Estimates and corresponding CIs (95%) of phenotypic and genetic similarity between partners for BMI (body mass index), DPW (drinks per week), and CPD (cigarettes per day). Phenotypic similarity is examined by the effect of partner phenotype on ego’s phenotype controlling for partner’s and ego’s age and sex. Genetic similarity is examined by the effect of partner’s PGI on ego’s PGI controlling for partner’s and ego’s age and sex and ego’s 20 PCs. Outcomes are standardized as are the PGIs. Results are separated by dataset: HRS and ELSA. Meta-estimates combing the results from HRS and ELSA are also included. The observed genetic similarity is compared to a theoretical genetic similarity that can be expected based on the heritability of the outcome, the phenotypic similarity, and the ‘quality’ of the predictor (phenotypic correlation × squared effect of own PGI on own phenotype). Phenotypic CIs are robust to clustering within households

Figure 1 shows there is considerable similarity at the phenotypic level in health behavior but there is a much lower similarity at the genetic level. Correlations for observed health behaviors between married/cohabiting partners were modest to large (HRS, BMI *r =* 0.23, DPW *r =* 0.48, and CPD *r =* 0.33; ELSA, BMI *r =* 0.20, DPW *r =* 0.50, and CPD *r =* 0.25). To examine genetic similarity, we examine the correlation between ego’s and partner’s PGIs for each of these three health behaviors and outcomes, controlling for partner’s and ego’s age and sex and ego’s 20 first principal components. We first confirmed that the PGI were associated with their corresponding phenotypes. Standardized estimates of ego’s PGI were largest for BMI (HRS, *r =* 0.35, *p* < 0.001; ELSA, *r* = 0.36, *p* < 0.001), then DWP (HRS, *r* = 0.11, *p* < 0.001; ELSA, *r* = 0.16, *p* < 0.001), and then CPD (HRS, *r =* 0.06, *p* < 0.001; ELSA, *r =* 0.06, *p* < 0.001; see also supplementary material). The genetic correlation between partners is insignificant for all three health outcomes in the ELSA, but is significant for BMI and CPD in the HRS (BMI, *r* = 0.06, *p* < 0.001; CPD, *r* = 0.04, *p* = 0.001). What is more, the estimates for these two genetic similarities are significantly different from what would be expected under phenotypic assortative mating (see theoretical genetic in Fig. 1). Hence, we find some evidence for genetic assortative mating in BMI and CPD, but only in the HRS (US) and not in the ELSA (UK).

### Is the Partner’s Genome Associated with Health Behavior?

**Fig. 2:**
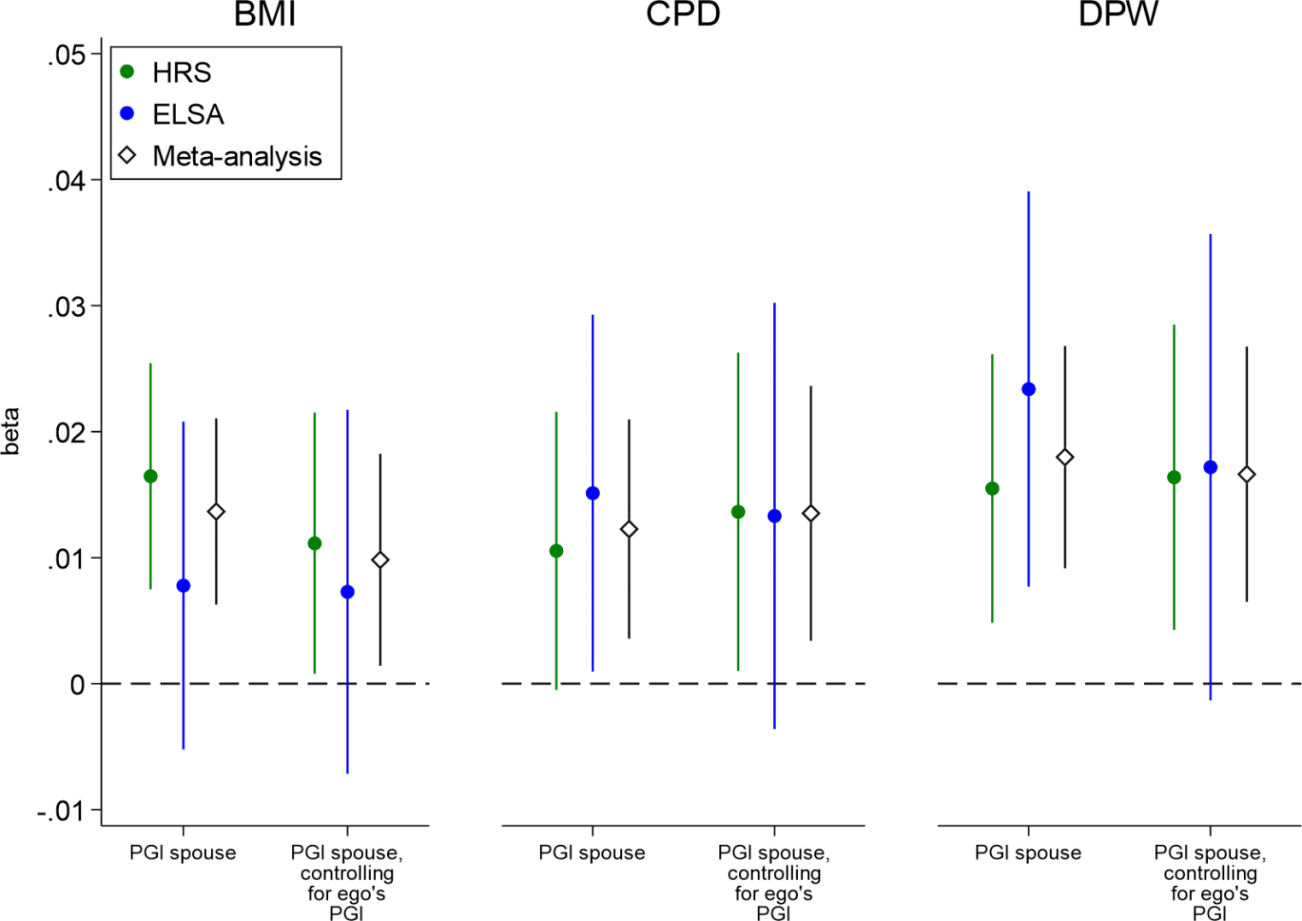
Social genetic effects of the partner conditioning on initial behavior of ego. Effect of partner PGI net of one’s own initial level of each outcome for BMI (body mass index), DPW (drinks per week), and CPD (cigarettes per day) on associated outcomes (time-varying) with socio-demographic controls and PCs of the partner. We also show estimates additionally controlling for ego’s PGI and PCs. Outcomes are standardized as are the PGIs. The figures show the effect per data source (HRS and ELSA) and a meta-analytic effect that combines both data sources. CIs (95%) are robust to clustering within individuals and households

Estimates of social genetic effects of the partner are summarized in Fig. 2 (detailed estimates are reported in supplementary material Table S3). We show estimates separately for HRS and ELSA and we report combined meta-analytic estimates (inverse variance method) that maximize the statistical power. We report the effects of the partner PGI controlling for ego’s first observed scores for a phenotype (BMI/CPD/DPW respectively), thereby directly controlling for observed phenotypic selection. This approach should be seen as a conservative test of our hypothesis of social genetic effects. By conditioning on previous behavior, we are effectively examining inter-individual *change* in health behavior during the period of observation. And by using the HRS and ELSA data, we examine change for relatively old samples of couples who are in long-lasting relationships. Nevertheless, we observe positive social genetic effects of the partner on ego’s behavior for each of the three health outcomes.

When we examine effects per data source (HRS and ELSA), we see that the point estimates are very similar between the HRS and ELSA, but the confidence intervals are much narrower in the HRS than in the ELSA. The HRS has more participants and a longer period of observation compared to ELSA (see supplementary material for details), which increases statistical power compared to ELSA. In all but one model (CPD without controlling for ego PGI), the social genetic effects are significant in the HRS. In the ELSA, all effects are also in the expected positive direction, although the smaller number of participants and shorter observation window leads to lower power to detect significant effects. Most importantly, the meta-analytic estimates combining the data from the HRS and ELSA show that the social genetic effects are significant for all three health outcomes, regardless of whether we control for ego’s PGI or not. Hence, we find robust evidence for social genetic effects when we combine data sources to obtain sufficient power. In contrast, our negative control – the social genetic effect for height – shows a null effect even when we combine data sources (Figure S4). Sex-stratified analyses are provided in the supplementary material (Figure S2 and Table S4-5). They provide little evidence for differences in social genetic effects by sex. Only for CPD, there is some evidence suggesting stronger social genetic effects for men than women.

The results of the IV analyses suggest that the partner’s health behaviors and outcomes are a plausible mechanism for our observed social genetic effects (Table S8). That is, whenever we observe a significant social genetic effect in our main analyses, we also observe a significant effect in the IV analyses where the partner’s behavior is instrumented by the partner’s polygenic index. The results of the multilevel multivariate analyses (Table S12) and change score analyses (Table S9) corroborate our main analyses; we find evidence for social genetic effects, with generally larger effect sizes and smaller standard errors in the HRS than in the ELSA.

**Fig. 3:**
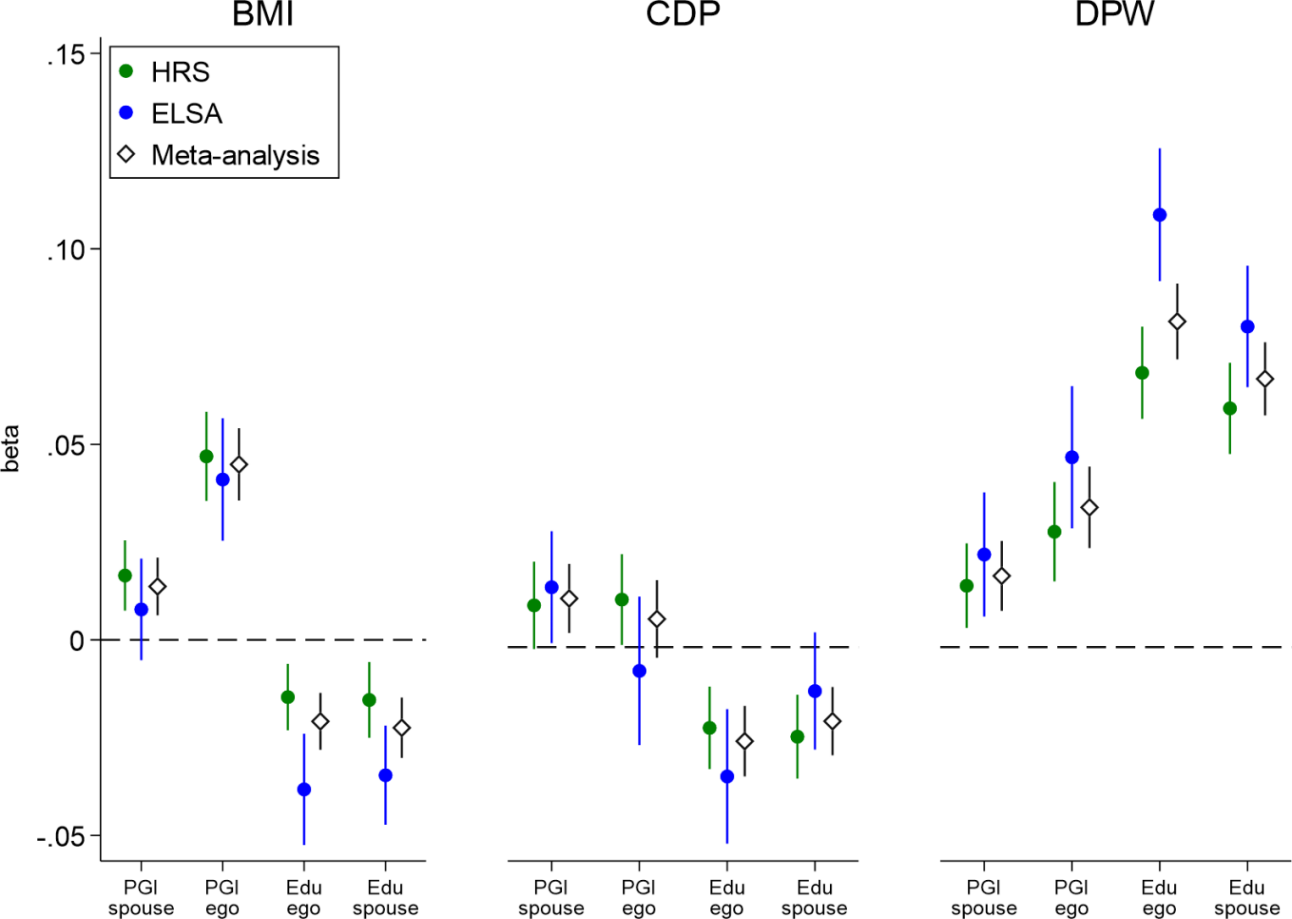
Effect size comparison of social genetic effects, direct genetic effects, and education effects. Estimates of direct genetic (PGI ego), social genetic (PGI spouse), and education effects of ego (Edu ego) and partner (Edu spouse) on BMI (body mass index), DPW (drinks per week), and CPD (cigarettes per day). Each effect is estimated from a separate model controlling for ego’s initial level of each outcome and socio-demographic controls. Note that the PGI spousal estimates are identical to those of Fig. 2 (PGI spouse, not controlled for ego’s PGI). Outcomes are standardized as are the PGIs. The figure shows the effects per data source (HRS and ELSA) and a meta-analytic effect that combines both data sources (inverse variance method). CIs (95%) are robust to clustering within individuals and households

To put some perspective on the effect sizes of the estimated social genetic effects, we compare the effect of partner’s PGI to the effect of ego’s own PGI and to ego’s and partner’s education level. Again, we use random effect regression models controlling for ego’s first observed scores for each phenotype (BMI/CPD/DPW respectively) and sex, age and age^2^ of both partners, sex interacted with age and age^2^, and relationship duration (Fig. 3, full details in supplementary material Table S6). Previous research shows that education is strongly linked to BMI, CPD, and DPW (more educated people tend to be less overweight, to smoke less, but to drink more; Cutler and Lleras-Muney 2010; Dupre 2008; Monden 2007; Nilsen et al. 2012; Pampel et al. 2015), making it a useful additional benchmark for assessing (absolute) effect sizes. For BMI, we find that the social genetic effect is about one-third of the direct effect of one’s own PGI on BMI. The social genetic effect is about two-thirds of the estimate for ego’s and the partner’s education level. For CPD, the social genetic effect is on par with the direct effect of one’s own PGI and similar to the estimate for one’s own and partner’s education. For DPW, the social genetic effect is about one-half of the direct effect of one’s own PGI. The size of the social genetic effect is about a quarter of the effect of ego’s and partner’s education level. In sum, the relative effect size of the social genetic effect is highest for CPD, but is also meaningful for BMI and DPW. For all comparisons we find that the social genetic effect is substantial; ranging from a quarter in absolute size as a minimum to being of equal size as that of the direct effect of one’s own genes, one’s own education, or the partner’s education.

## Discussion

We examined genetic similarity and social genetic effects among married and cohabiting partners for three widely studied health behaviors and outcomes (BMI, drinking, and smoking) using genome-wide data of couples in the HRS and ELSA, large nationally representative samples of the American population (HRS) and the English population (ELSA) aged 50 and older. We confirm previous research showing the existence of limited genetic similarity between partners for BMI (Conley et al. 2016) and we provide novel estimates of partner genetic similarity for drinking and smoking behavior. As expected, phenotypic similarity is much higher than genetic similarity. We find some evidence for genetic assortative mating in BMI and CPD, but only in the HRS. For drinking, we find no evidence for genetic assortative mating neither in the HRS nor in the ELSA. We do observe robust social genetic effects, which offers an alternative genetically rooted explanation for the large phenotypic partner similarity in healthy lifestyle. People are more overweight, drink more, and smoke more, if they have partners with higher polygenic indexes for these behaviors and outcomes.

Social genetic effects are generally lower in magnitude than direct genetic effects and education effects based on similar statistical models, but not much lower. For smoking, social genetic effects are on par with direct genetic effects. In general, the difference between direct genetic effects and social genetic effects is smaller for smoking and drinking than for BMI, which provides some suggestive evidence that substance use is driven relatively more so by social contagion than BMI. This is in line with studies suggesting that substance use is often a social activity with strong influences from one’s social network and partners in particular (Cooper et al. 2015; Votaw and Witkiewitz 2021). Altogether, our results suggest an important role for the partner’s genome.

Social genetic effects form a novel way to study the partner’s influence and the couple environment for health. This approach improves upon the reflection problem and reverse causality that hampers studies of social influence because the partner’s genotype, as opposed to his/her phenotype, is immune to influences from the couple environment and direct influences of ego once a partnership has formed. In contrast to prior research on social genetic effects, we used longitudinal data to control for phenotypic assortative mating and assess whether a partner’s genotype influences the change in ego’s phenotype over time. In doing so, we provided estimates for social genetic effects in health behaviors and outcomes while explicitly accounting for assortative mating.

Our paper revealed genetic evidence of social contagion within couples, but has some limitations that we hope future research will confront. First, the analyses were based on the HRS and ELSA, which are samples of older adults who may be set in their ways and hard to influence in their behavior. Indeed, evidence suggests that convergence between partners in smoking and drinking is most evident during the period before marriage/cohabitation, although convergence in physical activity is observed throughout life (Ask et al. 2012). We suspect that social genetic effects will be larger for younger populations, but this remains to be studied. Our results may thus be seen as conservative estimates for social genetic effects. Although inter-partner influences are potentially weakened among older adults, they are still often present and detectable. Studies find that health behaviors of one partner still influence changes in health behaviors of the other partner among middle-aged and older adults (Hoppmann and Gerstorf 2009; Windle and Windle 2014; Cobb et al. 2016; Ukai et al. 2022). In this regard, our results are consistent with the literature showing small but ongoing inter-partner health influences in older adults who have been together for relatively long periods of time.

Indeed, aging brings particular and new health issues that can lead to ongoing partner influence. For example, if a partner develops lung problems due to prolonged smoking and has to stop smoking on doctor’s orders, this might affect the smoking behavior of the other partner (e.g., out of consideration for one’s partner’s lung problems). Similar mechanisms can be thought of for BMI and drinking. For example, if one partner stops drinking because of liver damage accumulated over the years, this might cause the other partner to stop as well; and if one partner starts exercising because of weight gained over the years, this might induce the other partner to start exercising. Moreover, nowadays there is more information on, and awareness of, the negative effects of unhealthy lifestyles compared to the previous decades (Stead et al. 2019). This means that older couples (some of whom entered the sample in the 90s) over the years may have become more aware of the potential negative consequences of unhealthy lifestyles, giving them potential reasons to change their behavior even at an older age.

Over time, couples whose lifestyles deviate from one another may be more likely to separate/divorce (Torvik et al. 2013, 2015) and were less likely to enter our sample. Moreover, partners who are less aligned with each other may be less likely to both (continue to) participate in long-running studies like the HRS and ELSA, and as such cannot be included in analyses of partner influence. These are inherent difficulties in studying inter-partner influence. Two of our results shed light on the severity of this issue to some extent. First, we can compare the analyses with and without controlling for ego’s own polygenic index. By including ego’s own polygenic index, we require couples in which both partners participated in the genetic part of the study. Without including ego’s own polygenic index, we can include couples in which only one of the two partners participated in the genetic part of the study. In Fig. 2, we see that the significance and magnitude of our observed social genetic effects do not appreciably differ between analyses with and without controls for ego’s polygenic index. This provides some suggestive indication that we still find evidence for social genetic effects in potentially less aligned couples (in which only one partner participated in the genetic part of the study). Second, we found that observed social genetic effects remain when controlling for the baseline difference between partners in the phenotype of interest (BMI, drinking, or smoking). Although these analyses cannot conclusively show that social genetic effects would still appear in less aligned couples who were left out due to missing data, they provide some reassurance.

In addition, the results may refer to a relatively healthy subset of couples (Domingue et al. 2017), as we limited the sample to couples where at least one individual was genotyped, but genotyping took place took after the studies commenced. This means that the individuals (and the partnership) had to survive till that time. Second, we used the most up-to-date GWAS to create polygenic indexes for BMI, drinking, and smoking (Yengo et al. 2018b; Liu et al. 2019; Becker et al. 2021). These captured a large fraction of the SNP-based heritability for the studied health behaviors, but still a part is missing. PGIs are likely to become more predictive as larger GWAS come out and so the potential to detect social genetic effects will increase.

We find positive social genetic effects for all three different health behaviors, which supports the notion of social contagion of healthy lifestyle among couples, but we largely refrained from making statements about differences in the partner’s influence between specific behaviors. It would be difficult to do so, as the size of a social genetic effect not only depends on social factors, but also on the heritability of an outcome and how well we can estimate the genetic influences and how these differ among the three outcomes. Third, we limited the analyses to individuals of European descent because the polygenic indexes were based on GWAS of people of European descent and are therefore less predictive in other ancestries. This choice limits the generalizability of our findings. Fourth, one’s PGI can be seen as an indirect measure of parental genotypes, so the social genetic effects of the partner may also reflect possible social genetic effects of parents-in-law (through socialization). However, maybe such effects should not be ruled out, because interpreting social genetic effects as the effects of fixed predispositions of a partner on ego would include such socialization effects. Finally, we studied the three health behaviors in isolation and did not examine cross-trait effects. It is plausible that there may be cross-trait social genetic effects given large phenotypic and genotypic correlations between BMI, drinking, and smoking (Liu et al. 2019) and the possibility that genes have ‘pleiotropic effects’ (having effects on multiple phenotypes; Lee et al. 2012; Visscher et al. 2017). A suggestion to study social influence effects using genes as instrumental variables forms another approach that may give insight here, but suffers from stricter assumptions (O’Malley et al. 2014).

We focused on health behavior in married and cohabiting relationships. There is no reason why social genetic effects would be limited to this tie only. The environments (worksites, neighborhoods, schools, households, etc.) we navigate in life are populated by others who shape and mold these environments. These environments can be seen as genetic landscapes, which may give insights into the role of colleagues (Christakis and Fowler 2013), neighbors (Daw et al. 2014), and other household members (Guo et al. 2015; Rauscher et al. 2015). Moreover, besides health behaviors, other phenotypes could be susceptible to social genetic effects, for instance, related to mental health and well-being (Okbay et al. 2016).

The results contribute to a broader understanding of how health behaviors are molded and highlight the importance of people’s social surroundings. Why do some people keep smoking despite widespread knowledge of its harmful effects? The partner is likely important, but is that because of health selection which clusters health and wealth, because of shared environmental influences that affect both partners, or because partners reinforce each other and keep unhealthy behavior locked in place? Our results suggest that this last reason is likely, and suggests an important role of the partner. Furthermore, this study has illustrated how genetic variation within and between couples can help to overcome difficulties in making causal inferences regarding the role of the partner for health. Future applications could widen the evidential base of health prevention programs and/or medical care, as positive partner influence may lead to (unintended) positive spill-over effects on partners. These findings highlight the potential of targeting health interventions at couples, at partners of people who have already been identified as being at high risk, and at people most susceptible to social influence of the partner.

Previous research suggests that marriage and cohabitation can bring important health benefits, e.g., in the form of self-reported health, happiness, and reduced mortality (Johnson et al. 2000; Robards et al. 2012; Amato 2015). Our results nuance these findings, and suggest that whether a marriage or partnership brings health benefits depends on the partner’s (genetic) tendencies for a healthy lifestyle. Entering a marriage or partnership with a partner with a higher genetic predisposition for unhealthy behaviors and outcomes may lead to health disadvantages rather than benefits, or counteract the other health benefits of cohabitation and partnerships in general. Understanding the potential of health spillovers within couples can guide future efforts to limit negative spillovers and leverage partner influence for positive spillovers.

## Electronic Supplementary Material

Below is the link to the electronic supplementary material.

Supplementary Material 1

## Data Availability

GWAS summary data that inform the PGI are publicly available from the GSCAN and Polygenic Index Repository websites/original publications. HRS phenotypic data are publicly available via the HRS website (https://hrs.isr.umich.edu). ELSA data are available via the UK data archive https://www.data-archive.ac.uk/.
