# Supplementary material for: Partners in Health: Investigating Social Genetic Effects Among Married and Cohabiting Couples
Source: Behav Genet. 2023 Jun 7;53(4):348–58. doi: 10.1007/s10519-023-10147-w (PMC10276063; doi:10.1007/s10519-023-10147-w)
Supplement: Supplementary file 1 — Supplementary Material 1 [file 10519_2023_10147_MOESM1_ESM.docx]

**Supplementary Material for:**

Partners in health: Investigating social genetic effects among married and cohabiting couples

Kasper Otten^a^ & Jornt Mandemakers^a,b^

^a^ Utrecht University, Utrecht, the Netherlands

^b^ Atlas Research, Amsterdam, the Netherlands

Contents

1. Polygenic index and phenotypes
2. Phenotypic and genotypic partner similarity, Figure S1
3. Descriptive statistics, Table S1 and S2
4. Coefficient estimates underlying Figure 2, Table S3
5. Sex-stratified analyses. Figure S2, Tables S4 and S5
6. Coefficient estimates underlying Figure 3, Table S6
7. Sensitivity checks, Figure S3
8. Height as negative control, Figure S4 and Table S7
9. Instrumental variable analyses, Table S8
10. Analyses using change scores, Table S9
11. Social genetic effects by years between current and baseline observation, Table S10
12. Analyses controlling for baseline difference in behavior between ego and partner, Table S11
13. Multilevel multivariate analyses, Table S12
14. **Polygenic index and phenotypes**

The PGI for BMI from the Polygenic Index Repository was based on earlier GWAS (Locke et al. 2015; Yengo et al. 2018). It accounts for ~12.7% of the variation in BMI using SNPs at p<.0001 (Becker et al. 2021). That is about half of the proportion that is believed to be due to additive genetic factors (SNP heritability=~24.3%). The PGI for smoking (cigarettes per day, CPD) was based on the largest GWAS using data for 1.2 million people and accounts for about ~4% of the variation in smoking behavior (SNP heritability=~8.0%; Liu et al. 2019). The PGI for DPW accounts for ~1.3% in drinking behavior (SNP heritability is ~5.5%; Becker et al. 2021).

Using the analytical samples, the PGI’s showed clear associations with their respective outcomes. An increase of 1 S.D. (standard deviation) in the PGI for BMI was associated with a 1.9 point increase in BMI in the HRS (incremental r^2^=.12, net of age, age squared, sex, wave, and 20 PCs), and increases of 1 S.D. in the PGI for CPD and DPW were associated with .44 more cigarettes per day (r^2^=.004) and 1.1 more drinks per week (r^2^=.012) respectively. In the ELSA increases of 1 S.D. in the PGIs were associated with a 1.7 point increase in BMI (r^2^=.13), with .36 more cigarettes per day (r^2^=.003) and 1.2 more drinks per week (r^2^=.03) respectively.

1. **Phenotypic and genotypic partner similarity, Figure S1**

Figure S1 presents partner similarity on the phenotypic and genetic level for the HRS and ELSA. As reported in the main body of the paper, we find strong similarity at the phenotypic level, but weak similarity on the genetic level for all three outcomes: body mass index (BMI), alcoholic drinks per week (DPW), and cigarettes per day (CPD).

Figure S1. Phenotypic and genotypic partner similarity for BMI (weight/height^2^), CPD (cigarettes/day)), and DPW (ln(drinks/week)) in the HRS (left) and ELSA (right).

| **HRS** | **ELSA** |
| --- | --- |
| 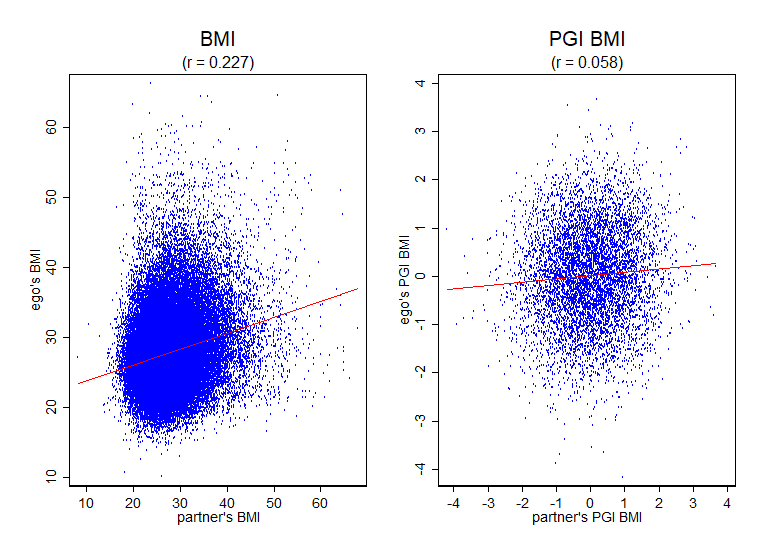 | 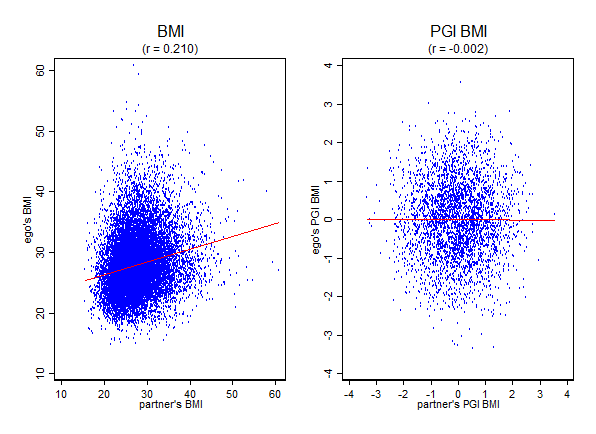 |
| 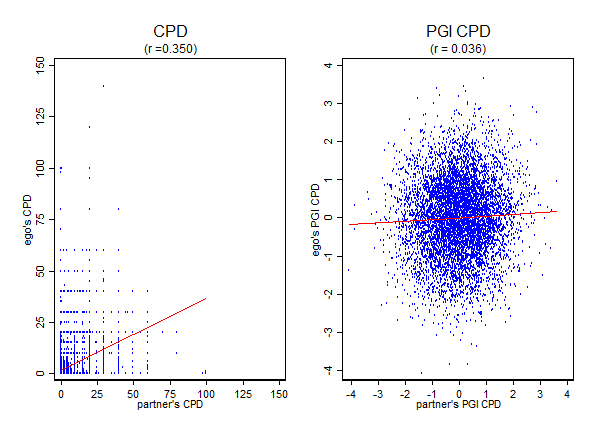 | 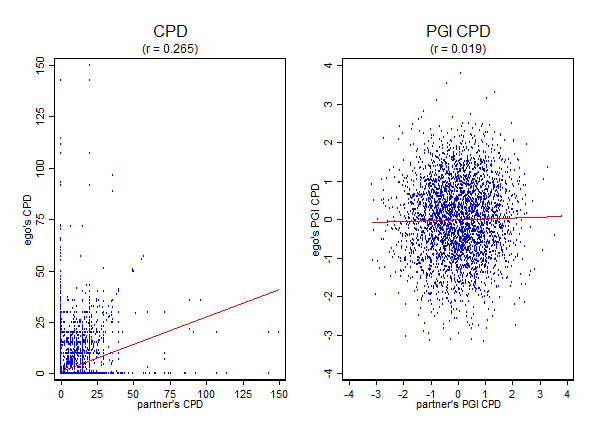 |
| 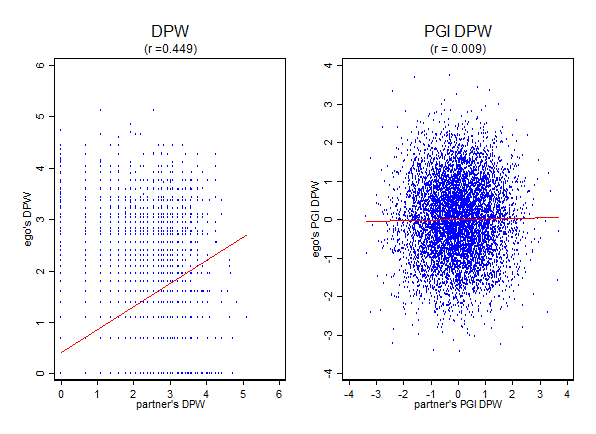 | 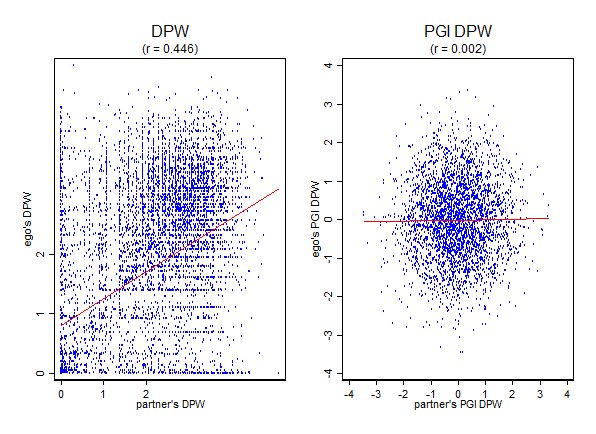 |

Note: For the phenotypic scatterplots/correlations all observations were used, for the genotypic scatterplots/correlations one observation per individual was used.

1. **Descriptive statistics, Table S1 and S2**

The descriptive statistics are presented in Table S1 (HRS) and Table S2 (ELSA). As is evident both the American HRS and the English ELSA are comprised of older people, the average age is 65. Most relationships are long-lasting, the average duration is 34 years in the HRS and 31 in the ELSA, which is likely due to the longer follow-up in the HRS. The average BMI is relatively high in both samples (>25 is commonly considered overweight), and both drinking and smoking are common, but Americans tend to smoke a bit more and drink much less than the English (respondents consume on average 3 drinks/week and 2 cigarettes/day in the HRS, whereas this is on average 6.7 drinks/week and 1.5 cigarettes/day in the ELSA). Comparing husbands to wives, we see in both studies that on average husbands are older (~2-3 years) and consume about double the number of drinks/week as their counterparts (~2-4 drinks more per week), but do not substantially smoke more (~1/4 cigarettes more per day only in the HRS). Husbands are more overweight than their wives in the HRS (~1 BMI point), but not in the ELSA.

Table S1. HRS Descriptive statistics (means (s.d.)).

|  |  | BMI |  |  | CPD |  |  | DPW |  |
| --- | --- | --- | --- | --- | --- | --- | --- | --- | --- |
|  | pooled | husbands | wives | pooled | husbands | wives | pooled | husbands | wives |
| BMI (weight/height^2^) | 27.94 | 28.43 | 27.46 |  |  |  |  |  |  |
|  | (5.38) | (4.75) | (5.89) |  |  |  |  |  |  |
| CPD (cigarettes/day) |  |  |  | 2.06 | 2.27 | 1.87 |  |  |  |
|  |  |  |  | (6.83) | (7.50) | (6.11) |  |  |  |
| drinks/week |  |  |  |  |  |  | 3.05 | 4.10 | 2.07 |
|  |  |  |  |  |  |  | (6.47) | (7.90) | (4.54) |
| DPW |  |  |  |  |  |  | .75 | .91 | .60 |
| (ln(drinks+1/week)) |  |  |  |  |  |  | (1.01) | (1.11) | (.89) |
|  |  |  |  |  |  |  |  |  |  |
| initial outcome_ego, t=1_ | 27.29 | 28.01 | 26.59 | 3.53 | 4.07 | 3.02 | .77 | .97 | .58 |
|  | (5.00) | (4.35) | (5.47) | (9.25) | (10.38) | (7.99) | (1.01) | (1.12) | (.86) |
| age_ego_ | 64.22 | 65.51 | 62.97 | 64.20 | 65.51 | 62.95 | 64.97 | 66.22 | 63.80 |
|  | (8.19) | (7.62) | (8.52) | (8.19) | (7.62) | (8.52) | (8.07) | (7.55) | (8.37) |
| age_partner_ | 64.39 | 62.36 | 66.37 | 64.41 | 62.36 | 66.36 | 65.28 | 63.21 | 67.20 |
|  | (8.63) | (8.32) | (8.46) | (8.63) | (8.32) | (8.46) | (8.52) | (8.18) | (8.37) |
| male_ego_ | .49 |  |  | .49 |  |  | .48 |  |  |
|  | (.50) |  |  | (.50) |  |  | (.50) |  |  |
| PGI BMI_ego_^a^ | -.00 | .01 | -.02 |  |  |  |  |  |  |
|  | (1.00) | (.99) | (1.01) |  |  |  |  |  |  |
| PGI BMI_partner_ | -.00 | -.01 | .00 |  |  |  |  |  |  |
|  | (1.00) | (1.01) | (.99) |  |  |  |  |  |  |
| PGI CPD_ego_^a^ |  |  |  | -.00 | -.06 | .05 |  |  |  |
|  |  |  |  | (1.00) | (1.00) | (1.00) |  |  |  |
| PGI CPD_partner_ |  |  |  | -.00 | .05 | -.05 |  |  |  |
|  |  |  |  | (1.00) | (1.00) | (.99) |  |  |  |
| PGI DPW_ego_^a^ |  |  |  |  |  |  | .00 | -.02 | .02 |
|  |  |  |  |  |  |  | (1.00) | (1.01) | (.99) |
| PGI DPW_partner_ |  |  |  |  |  |  | -.00 | .02 | -.02 |
|  |  |  |  |  |  |  | (1.00) | (.99) | (1.00) |
| year of observation | 2005.95 | 2005.74 | 2006.17 | 2005.95 | 2005.74 | 2006.15 | 2007.59 | 2007.47 | 2007.70 |
|  | (6.99) | (7.02) | (6.95) | (6.98) | (7.02) | (6.94) | (5.94) | (5.96) | (5.92) |
| relationship length | 34.04 | 33.69 | 34.38 | 34.05 | 33.68 | 34.40 | 34.53 | 34.16 | 34.89 |
| (years) | (15.19) | (15.08) | (15.29) | (15.19) | (15.08) | (15.28) | (15.47) | (15.36) | (15.57) |
| *N* observations | 59325 | 29255 | 30070 | 60029 | 29289 | 30740 | 52023 | 25120 | 26903 |
| *N* individuals | 9522 | 4985 | 4537 | 9546 | 4987 | 4559 | 9140 | 4707 | 4433 |

^a^ reduced sample size for PGI of ego, *N*=48572 for BMI, *N*=49108 for CPD, *N*=43358 for DPW.

Table S2. ELSA Descriptive statistics (means (s.d.)).

|  |  | BMI |  |  | CPD |  |  | DPW |  |
| --- | --- | --- | --- | --- | --- | --- | --- | --- | --- |
|  | pooled | husbands | wives | pooled | husbands | wives | pooled | husbands | wives |
| BMI (weight/height^2^) | 28.09 | 28.05 | 28.13 |  |  |  |  |  |  |
|  | (4.93) | (4.23) | (5.48) |  |  |  |  |  |  |
| CPD (cigarettes/day) |  |  |  | 1.51 | 1.51 | 1.52 |  |  |  |
|  |  |  |  | (6.04) | (6.58) | (5.54) |  |  |  |
| drinks/week |  |  |  |  |  |  | 6.70 | 8.78 | 4.88 |
|  |  |  |  |  |  |  | (9.54) | (11.14) | (7.41) |
| DPW |  |  |  |  |  |  | 1.43 | 1.70 | 1.19 |
| (ln(drinks+1/week)) |  |  |  |  |  |  | (1.13) | (1.14) | (1.07) |
|  |  |  |  |  |  |  |  |  |  |
| initial outcome_ego, t=1_ | 27.61 | 27.72 | 27.51 | 2.45 | 2.58 | 2.33 | 1.84 | 2.24 | 1.50 |
|  | (4.57) | (3.82) | (5.16) | (6.91) | (7.36) | (6.49) | (1.16) | (1.13) | (1.07) |
| age_ego_ | 66.48 | 67.37 | 65.68 | 64.69 | 66.13 | 63.45 | 64.68 | 66.06 | 63.47 |
|  | (6.92) | (6.78) | (6.94) | (8.03) | (7.48) | (8.28) | (7.97) | (7.43) | (8.22) |
| age_partner_ | 66.84 | 65.34 | 68.21 | 65.51 | 64.34 | 66.52 | 65.49 | 64.27 | 66.55 |
|  | (7.32) | (6.92) | (7.41) | (7.71) | (7.19) | (8.00) | (7.68) | (7.15) | (7.96) |
| male_ego_ | .48 |  |  | .46 |  |  | .47 |  |  |
|  | (.50) |  |  | (.50) |  |  | (.50) |  |  |
| PGI BMI_ego_^a^ | .00 | .01 | .00 |  |  |  |  |  |  |
|  | (1.00) | (1.02) | (.99) |  |  |  |  |  |  |
| PGI BMI_partner_ | -.00 | -.01 | .01 |  |  |  |  |  |  |
|  | (1.00) | (.98) | (1.02) |  |  |  |  |  |  |
| PGI CPD_ego_^a^ |  |  |  | .00 | -.04 | .04 |  |  |  |
|  |  |  |  | (1.00) | (.98) | (1.01) |  |  |  |
| PGI CPD_partner_ |  |  |  | -.00 | .02 | -.02 |  |  |  |
|  |  |  |  | (1.00) | (1.01) | (.98) |  |  |  |
| PGI DPW_ego_^a^ |  |  |  |  |  |  | .00 | .01 | -.00 |
|  |  |  |  |  |  |  | (1.00) | (1.00) | (1.00) |
| PGI DPW_partner_ |  |  |  |  |  |  | .00 | -.00 | .01 |
|  |  |  |  |  |  |  | (1.00) | (1.00) | (1.00) |
| year of observation | 2010.63 | 2010.48 | 2010.77 | 2009.00 | 2008.93 | 2009.07 | 2009.07 | 2008.99 | 2009.14 |
|  | (4.97) | (4.96) | (4.98) | (4.91) | (4.90) | (4.91) | (4.93) | (4.94) | (4.92) |
| relationship length | 30.65 | 30.18 | 31.08 | 28.98 | 28.70 | 29.22 | 29.09 | 28.67 | 29.45 |
| (years) | (17.19) | (17.20) | (17.18) | (17.20) | (17.39) | (17.03) | (17.12) | (17.31) | (16.95) |
| *N* observations | 11728 | 5574 | 6154 | 25740 | 11925 | 13815 | 24179 | 11254 | 12925 |
| *N* individuals | 4311 | 2060 | 2251 | 4943 | 2383 | 2560 | 4911 | 2366 | 2545 |

^a^ reduced sample size for PGI of ego, *N*=9155 for BMI, *N*=18270 for CPD, *N*=17485 for DPW.

1. **Coefficient estimates underlying Figure 2, Table S3**

Table S3 presents the coefficients of the models underlying Figure 2 in the main text. The meta-analytic estimates (not depicted in Table S3) of Figure 2 were calculated using the inverse variance method (i.e. the weight given to each study is the inverse of the variance of the effect estimate).

Table S3. Random effects regression of BMI, DPW, CPD (t) (standardized) on the partner’s PGI conditioned on first observed BMI/DPW/CPD of ego in the HRS and ELSA.
1) Preferred model, 2) Model controlling for ego’s PGI, and 3) comparison model (specification as preferred model with same N as model controlling for ego’s PGI). Standard errors adjusted for clustering in individuals and households.

|  |  |  | **HRS** |  |  |  | **ELSA** |  |
| --- | --- | --- | --- | --- | --- | --- | --- | --- |
|  |  | (1) | (2) | (3) |  | (1) | (2) | (3) |
|  |  | preferred | ego PGI | comparison |  | preferred | ego PGI | comparison |
| **BMI** |  |  |  |  |  |  |  |  |
| PGI BMI_partner_ (std.) |  | .016^***^ | .011^*^ | .013^*^ |  | .008 | .007 | .006 |
|  |  | (.005) | (.005) | (.005) |  | (.007) | (.007) | (.007) |
| PGI BMI_ego_ (std.) |  |  | .046^***^ |  |  |  | .041^***^ |  |
|  |  |  | (.006) |  |  |  | (.008) |  |
| BMI_ego, t=1_ |  | .877^***^ | .862^***^ | .879^***^ |  | .952^***^ | .935^***^ | .951^***^ |
|  |  | (.008) | (.010) | (.009) |  | (.012) | (.015) | (.014) |
| BMI_ego, t=1_ * time |  | -.004^***^ | -.004^***^ | -.004^***^ |  | -.007^***^ | -.006^***^ | -.006^***^ |
|  |  | (.001) | (.001) | (.001) |  | (.001) | (.002) | (.002) |
| *N* observations |  | 59325 | 48572 | 48572 |  | 11728 | 9155 | 9155 |
| *N* individuals |  | 9522 | 6914 | 6914 |  | 4311 | 3262 | 3262 |
| *N* households |  | 5879 | 3477 | 3477 |  | 2729 | 1680 | 1680 |
| **CPD** |  |  |  |  |  |  |  |  |
| PGI CPD_partner_ (std.) |  | .011^#^ | .014^*^ | .014^*^ |  | .015^*^ | .013 | .013 |
|  |  | (.006) | (.006) | (.006) |  | (.007) | (.009) | (.009) |
| PGI CPD_ego_ (std.) |  |  | .012^*^ |  |  |  | -.006 |  |
|  |  |  | (.006) |  |  |  | (.010) |  |
| CPD_ego, t=1_ |  | .961^***^ | .957^***^ | .957^***^ |  | .887^***^ | .865^***^ | .864^***^ |
|  |  | (.022) | (.023) | (.023) |  | (.041) | (.057) | (.057) |
| CPD_ego, t=1_ * time |  | -.037^***^ | -.036^***^ | -.036^***^ |  | -.034^***^ | -.034^***^ | -.034^***^ |
|  |  | (.002) | (.002) | (.002) |  | (.003) | (.004) | (.004) |
| *N* observations |  | 60029 | 49108 | 49108 |  | 25740 | 18270 | 18270 |
| *N* individuals |  | 9546 | 6931 | 6931 |  | 4943 | 3305 | 3305 |
| *N* households |  | 5885 | 3478 | 3478 |  | 3311 | 1673 | 1673 |
| **DPW** |  |  |  |  |  |  |  |  |
| PGI DPW_partner_ (std.) |  | .015^**^ | .016^**^ | .017^**^ |  | .023^**^ | .017^#^ | .018^*^ |
|  |  | (.005) | (.006) | (.006) |  | (.008) | (.009) | (.009) |
| PGI DPW_ego_ (std.) |  |  | .029^***^ |  |  |  | .047^***^ |  |
|  |  |  | (.006) |  |  |  | (.009) |  |
| DPW_ego, t=1_ |  | .793^***^ | .806^***^ | .811^***^ |  | .661^***^ | .653^***^ | .663^***^ |
|  |  | (.009) | (.010) | (.010) |  | (.010) | (.013) | (.012) |
| DPW_ego, t=1_ * time |  | -.009^***^ | -.009^***^ | -.009^***^ |  | -.001 | -.001 | -.001 |
|  |  | (.001) | (.001) | (.001) |  | (.001) | (.001) | (.001) |
| *N* observations |  | 52023 | 43358 | 43358 |  | 24179 | 17485 | 17485 |
| *N* individuals |  | 9140 | 6866 | 6866 |  | 4911 | 3350 | 3350 |
| *N* households |  | 5584 | 3462 | 3462 |  | 3264 | 1703 | 1703 |
| controls |  | Yes | Yes | Yes |  | Yes | Yes | Yes |
| wave dummies |  | Yes | Yes | Yes |  | Yes | Yes | Yes |
| PCs_ego_ |  | No | Yes | No |  | No | Yes | No |
| PCs_partner_ |  | Yes | Yes | Yes |  | Yes | Yes | Yes |

Control variables were sex, age, age^2^ of both partners and interactions with age, age^2^, year of observation dummies, and relationship duration. PCs were the first 20 principal components.

^#^ *p* < .10, ^*^ *p* < .05, ^**^ *p* < .01, ^***^ *p* < .001

1. **Sex-stratified analyses (Figure S2 and Tables S4 and S5)**

Table S4 (HRS) and S5 (ELSA) show sex-stratified effects of partner PGI net of one’s own initial level of each outcome for BMI, DPW, and CPD on associated outcomes (time-varying) with socio-demographic controls and PCs of the partner. We also show estimates additionally controlling for ego’s PGI and PCs. Outcomes are standardized as are the PGIs. Figure S2 show the estimate per dataset (HRS and ELSA) and a meta-analytic estimate that combines both. CIs (95%) are robust to clustering within individuals and households. Meta-analytic estimates were calculated using the inverse variance method (i.e. the weight given to each study is the inverse of the variance of the effect estimate). Note that the social genetic effects differed significantly by sex only for CPD (based on pooled models; p<.05 for HRS; p<.10 for ELSA).

Figure S2. Social genetic effects of the partner conditioning on initial behavior of ego by sex.

Table S4 Random effects regression of BMI, DPW, CPD (t) (standardized) on the partner’s PGI conditioned on first observed BMI/DPW/CPD of ego in the HRS. Same model specifications as Table S3.

| **BMI** | men |  |  | women |  |  |
| --- | --- | --- | --- | --- | --- | --- |
|  | (4) | (5) | (6) | (7) | (8) | (9) |
|  | pref | ego PGI | comp. | pref | ego PGI | comp. |
| PGI BMI_partner_ (std.) | .021^***^ | .017^**^ | .018^**^ | .011 | .005 | .007 |
|  | (.005) | (.006) | (.006) | (.007) | (.008) | (.008) |
| PGI BMI_ego_ (std.) |  | .035^***^ |  |  | .058^***^ |  |
|  |  | (.007) |  |  | (.009) |  |
| BMI_ego, t=1_ | .876^***^ | .866^***^ | .881^***^ | .877^***^ | .859^***^ | .877^***^ |
|  | (.009) | (.012) | (.011) | (.011) | (.014) | (.013) |
| BMI_ego, t=1_ * time | -.004^**^ | -.004^**^ | -.004^**^ | -.004^***^ | -.004^***^ | -.004^***^ |
|  | (.001) | (.001) | (.001) | (.001) | (.001) | (.001) |
| *N* observations | 29255 | 23349 | 23349 | 30070 | 25223 | 25223 |
| *N* individuals | 4985 | 3435 | 3435 | 4537 | 3479 | 3479 |
| *N* households | 4865 | 3396 | 3396 | 4384 | 3440 | 3440 |
| **CPD** | men |  |  | women |  |  |
|  | (4) | (5) | (6) | (7) | (8) | (9) |
|  | pref | ego PGI | comp. | pref | ego PGI | comp. |
| PGI CPD_partner_ (std.) | .023^**^ | .027^**^ | .027^**^ | -.004 | -.002 | -.002 |
|  | (.008) | (.010) | (.010) | (.007) | (.008) | (.008) |
| PGI CPD_ego_ (std.) |  | .020^*^ |  |  | .005 |  |
|  |  | (.009) |  |  | (.007) |  |
| CPD_ego, t=1_ | .954^***^ | .916^***^ | .918^***^ | .980^***^ | 1.019^***^ | 1.019^***^ |
|  | (.030) | (.031) | (.031) | (.027) | (.029) | (.029) |
| CPD_ego, t=1_ * time | -.039^***^ | -.037^***^ | -.037^***^ | -.035^***^ | -.036^***^ | -.036^***^ |
|  | (.002) | (.002) | (.002) | (.002) | (.002) | (.002) |
| *N* observations | 29289 | 23371 | 23371 | 30740 | 25737 | 25737 |
| *N* individuals | 4987 | 3436 | 3436 | 4559 | 3495 | 3495 |
| *N* households | 4867 | 3397 | 3397 | 4403 | 3455 | 3455 |
| **DPW** | men |  |  | women |  |  |
|  | (4) | (5) | (6) | (7) | (8) | (9) |
|  | pref | ego PGI | comp. | pref | ego PGI | comp. |
| PGI DPW_partner_ (std.) | .016^#^ | .012 | .013 | .015^*^ | .020^**^ | .021^**^ |
|  | (.008) | (.010) | (.010) | (.007) | (.008) | (.008) |
| PGI DPW_ego_ (std.) |  | .033^***^ |  |  | .023^**^ |  |
|  |  | (.010) |  |  | (.008) |  |
| DPW_ego, t=1_ | .800^***^ | .814^***^ | .818^***^ | .785^***^ | .797^***^ | .802^***^ |
|  | (.011) | (.012) | (.012) | (.012) | (.013) | (.013) |
| DPW_ego, t=1_ * time | -.012^***^ | -.012^***^ | -.012^***^ | -.005^***^ | -.005^***^ | -.005^***^ |
|  | (.001) | (.001) | (.001) | (.001) | (.001) | (.001) |
| *N* observations | 25120 | 20581 | 20581 | 26903 | 22777 | 22777 |
| *N* individuals | 4707 | 3399 | 3399 | 4433 | 3467 | 3467 |
| *N* households | 4617 | 3369 | 3369 | 4317 | 3435 | 3435 |
| controls | Yes | Yes | Yes | Yes | Yes | Yes |
| wave dummies | Yes | Yes | Yes | Yes | Yes | Yes |
| PCs_ego_ | No | Yes | No | No | Yes | No |
| PCs_partner_ | Yes | Yes | Yes | Yes | Yes | Yes |

Controls variables were sex, age, age^2^ of both partners, all interacted with sex, and relationship duration.

^#^ *p* < .10, ^*^ *p* < .05, ^**^ *p* < .01, ^***^ *p* < .001

Table S5 Random effects regression of BMI, DPW, CPD (t) (standardized) on the partner’s PGI conditioned on first observed BMI/DPW/CPD of ego in the ELSA. Same model specifications as Table S3.

| **BMI** | men |  |  | women |  |  |
| --- | --- | --- | --- | --- | --- | --- |
|  | (4) | (5) | (6) | (7) | (8) | (9) |
|  | pref | ego PGI | comp. | pref | ego PGI | comp. |
| PGI BMI_partner_ (std.) | .010 | .008 | .009 | .005 | .005 | .004 |
|  | (.009) | (.009) | (.009) | (.010) | (.011) | (.011) |
| PGI BMI_ego_ (std.) |  | .044^***^ |  |  | .034^**^ |  |
|  |  | (.009) |  |  | (.012) |  |
| BMI_ego, t=1_ | .949^***^ | .936^***^ | .957^***^ | .955^***^ | .935^***^ | .947^***^ |
|  | (.014) | (.016) | (.015) | (.012) | (.015) | (.014) |
| BMI_ego, t=1_ * time | -.004^***^ | -.004^***^ | -.004^***^ | -.009^***^ | -.008^***^ | -.008^***^ |
|  | (.001) | (.001) | (.001) | (.001) | (.001) | (.001) |
| *N* observations | 5574 | 4475 | 4475 | 6154 | 4680 | 4680 |
| *N* individuals | 2060 | 1604 | 1604 | 2251 | 1658 | 1658 |
| *N* households | 2060 | 1604 | 1604 | 2251 | 1658 | 1658 |
| **CPD** | men |  |  | women |  |  |
|  | (4) | (5) | (6) | (7) | (8) | (9) |
|  | pref | ego PGI | comp. | pref | ego PGI | comp. |
| PGI CPD_partner_ (std.) | .028^*^ | .032^*^ | .031^*^ | .007 | -.003 | -.003 |
|  | (.012) | (.014) | (.014) | (.009) | (.011) | (.011) |
| PGI CPD_ego_ (std.) |  | -.025^#^ |  |  | .006 |  |
|  |  | (.014) |  |  | (.011) |  |
| CPD_ego, t=1_ | .825^***^ | .813^***^ | .810^***^ | .955^***^ | .914^***^ | .915^***^ |
|  | (.016) | (.021) | (.021) | (.013) | (.015) | (.015) |
| CPD_ego, t=1_ * time | -.034^***^ | -.034^***^ | -.034^***^ | -.034^***^ | -.033^***^ | -.033^***^ |
|  | (.001) | (.002) | (.002) | (.001) | (.001) | (.001) |
| *N* observations | 11925 | 8878 | 8878 | 13815 | 9392 | 9392 |
| *N* individuals | 2383 | 1637 | 1637 | 2560 | 1668 | 1668 |
| *N* households | 2383 | 1637 | 1637 | 2560 | 1668 | 1668 |
| **DPW** | men |  |  | women |  |  |
|  | (4) | (5) | (6) | (7) | (8) | (9) |
|  | pref | ego PGI | comp. | pref | ego PGI | comp. |
| PGI DPW_partner_ (std.) | .031^*^ | .025^#^ | .025^#^ | .017^#^ | .010 | .013 |
|  | (.012) | (.014) | (.014) | (.010) | (.012) | (.012) |
| PGI DPW_ego_ (std.) |  | .047^**^ |  |  | .044^***^ |  |
|  |  | (.014) |  |  | (.012) |  |
| DPW_ego, t=1_ | .611^***^ | .606^***^ | .615^***^ | .722^***^ | .713^***^ | .722^***^ |
|  | (.016) | (.019) | (.018) | (.014) | (.017) | (.017) |
| DPW_ego, t=1_ * time | .002 | .002 | .002 | -.005^***^ | -.005^***^ | -.005^***^ |
|  | (.001) | (.002) | (.002) | (.001) | (.001) | (.001) |
| *N* observations | 11254 | 8550 | 8550 | 12925 | 8935 | 8935 |
| *N* individuals | 2366 | 1662 | 1662 | 2545 | 1688 | 1688 |
| *N* households | 2366 | 1662 | 1662 | 2545 | 1688 | 1688 |
| controls | Yes | Yes | Yes | Yes | Yes | Yes |
| wave dummies | Yes | Yes | Yes | Yes | Yes | Yes |
| PCs_ego_ | No | Yes | No | No | Yes | No |
| PCs_partner_ | Yes | Yes | Yes | Yes | Yes | Yes |

Controls variables were sex, age, age^2^ of both partners, all interacted with sex, and relationship duration.

^#^ *p* < .10, ^*^ *p* < .05, ^**^ *p* < .01, ^***^ *p* < .001

1. **Coefficient estimates underlying Figure 3, Table S6**

Meta-analytic estimates of Figure 3 were calculated using the inverse variance method (i.e. the weight given to each study is the inverse of the variance of the effect estimate).

Table S6. Random effects regression of BMI, DPW, CPD (t) (standardized) on X (either ego’s PGI BMI/DPW/CPD, partners’ PGI BMI/DPW/CPD, ego’s education in years or the partner’s education in years) conditioned on first observed BMI/DPW/CPD of ego in the HRS and ELSA. Standard errors adjusted for clustering in individuals and households.

|  | **HRS** |  |  |  | **ELSA** |  |  |  |
| --- | --- | --- | --- | --- | --- | --- | --- | --- |
|  | PGI partner | PGI ego | Education ego | Education partner | PGI partner | PGI ego | Education ego | Education partner |
| **BMI** |  |  |  |  |  |  |  |  |
| X^a^ | .016*** | .047*** | -.015*** | -.015** | .008 | .041^***^ | -.038^***^ | -.035^***^ |
|  | (.005) | (.006) | (.004) | (.005) | (.007) | (.008) | (.007) | (.006) |
| BMI_ego, t=1_ | .877*** | .863*** | .877*** | .877*** | .952^***^ | .935^***^ | .950^***^ | .949^***^ |
|  | (.008) | (.010) | (.008) | (.008) | (.012) | (.015) | (.012) | (.012) |
| BMI_ego, t=1_ * time | -.004*** | -.004*** | -.004*** | -.004*** | -.007^***^ | -.006^***^ | -.007^***^ | -.007^***^ |
|  | (.001) | (.001) | (.001) | (.001) | (.001) | (.002) | (.001) | (.001) |
| *N* observations | 59325 | 48572 | 59325 | 59325 | 11728 | 9155 | 11362 | 11370 |
| *N* individuals | 9522 | 6914 | 9522 | 9522 | 4311 | 3262 | 4162 | 4161 |
| *N* households | 5879 | 3477 | 5879 | 5879 | 2729 | 1680 | 2684 | 2696 |
| **CPD** |  |  |  |  |  |  |  |  |
| X^a^ | .011# | .012* | -.020*** | -.023*** | .015* | -.006 | -.033*** | -.011 |
|  | (.006) | (.006) | (.005) | (.005) | (.007) | (.010) | (.009) | (.008) |
| CPD_ego, t=1_ | .961*** | .958*** | .958*** | .958*** | .887*** | .866*** | .885*** | .895*** |
|  | (.022) | (.023) | (.022) | (.022) | (.041) | (.057) | (.042) | (.041) |
| CPD_ego, t=1_ * time | -.037*** | -.036*** | -.037*** | -.037*** | -.034*** | -.034*** | -.035*** | -.036*** |
|  | (.002) | (.002) | (.002) | (.002) | (.003) | (.004) | (.003) | (.003) |
| *N* observations | 60029 | 49108 | 60029 | 60029 | 25740 | 18270 | 24944 | 24927 |
| *N* individuals | 9546 | 6931 | 9546 | 9546 | 4943 | 3305 | 4774 | 4753 |
| *N* households | 5885 | 3478 | 5885 | 5885 | 3311 | 1673 | 3251 | 3245 |
| **DPW** |  |  |  |  |  |  |  |  |
| X^a^ | .015** | .029*** | .069*** | .060*** | .023** | .048*** | .109*** | .081*** |
|  | (.005) | (.006) | (.006) | (.006) | (.008) | (.009) | (.009) | (.008) |
| DPW_ego, t=1_ | .793*** | .810*** | .789*** | .790*** | .661*** | .655*** | .646*** | .654*** |
|  | (.009) | (.010) | (.009) | (.009) | (.010) | (.013) | (.011) | (.010) |
| DPW_ego, t=1_ * time | -.009*** | -.009*** | -.009*** | -.010*** | -.001 | -.001 | -.001 | -.001 |
|  | (.001) | (.001) | (.001) | (.001) | (.001) | (.001) | (.001) | (.001) |
| *N* observations | 52023 | 43358 | 52023 | 52023 | 24179 | 17485 | 23427 | 23409 |
| *N* individuals | 9140 | 6866 | 9140 | 9140 | 4911 | 3350 | 4745 | 4726 |
| *N* households | 5584 | 3462 | 5584 | 5584 | 3264 | 1703 | 3208 | 3203 |

^a^ = either ego’s PGI BMI/DPW/CPD, partners’ PGI BMI/DPW/CPD, ego’s education in years or the partner’s education in years, see labelled column.

Control variables were sex, age, age^2^ of both partners, all interacted with sex, and relationship duration.

^#^ *p* < .10, ^*^ *p* < .05, ^**^ *p* < .01, ^***^ *p* < .001

1. **Sensitivity checks, Figure S3**

To assess the sensitivity of our results to outliers and sample selection criteria, we re-estimated our social genetic effect models (Model 1 and 2 in Table S3), but with the following differences:

- 1. BMI;
     1. Removing persons with bottom and top BMI scores (BMI < 20 and BMI > 40).
  2. CPD
     1. Removing heavy smokers (equivalent to a pack of 20 cigarettes or more a day).
     2. Removing persons that never reported to have smoked.
  3. DPW
     1. Removing heavy drinkers (≥ 28 drinks/week in the ELSA and > 14 in the HRS, which is equivalent to the top ~5% in the ELSA/HRS).
     2. Removing persons that never reported having drunk alcoholic beverages.

For ease of comparison, we show the main results on the uttermost left panel, and the restricted samples to the right. The results are presented in Figure S3A-C. The subheaders indicate which sensitivity check applies.

Figure S3A. Sensitivity check BMI

Figure S3B. Sensitivity check CPD

Figure S3C. Sensitivity check DPW

1. **Height as negative control, Figure S4 and Table S7**

We use the most recently available height PGI available in the ELSA and HRS datasets. The PGI provided in the ELSA dataset is based on a 2014 GWAS by the Genetic Investigation of ANthropometric Traits (GIANT) consortium (Wood et al. 2014), the PGI provided in the HRS dataset is based on a GWAS meta-analysis by the GIANT consortium (Yengo et al. 2018). Details on the derivation of these PGIs are available from the HRS and ELSA (Ajnakina and Steptoe 2019; Ware et al. 2021). We first confirmed that the PGIs were associated with their corresponding phenotypes in both datasets. Controlling for age, sex, and the first 20 PCs, we find a clear effect of ego’s PGI on the phenotype (HRS, *r* = .29, *p* < .001; ELSA, *r* = .24, *p* < .001). We furthermore find a significant phenotypic correlation between partners (HRS, *r* = .17, *p* < .001; ELSA, *r* = .25, *p* < .001) and a significant correlation between the PGIs of the partners (HRS, *r* = .04, *p* < .001; ELSA, *r* = .04, *p* = .01), controlling for partner’s and ego’s age and sex and ego’s 20 first principal components.

Estimates of social genetic effects of the partner are summarized in Figure S4, detailed estimates are reported in Table S7. We show estimates separately for HRS and ELSA and we report combined meta-analytic estimates that maximize the statistical power (using the inverse variance method, i.e. the weight given to each study is the inverse of the variance of the effect estimate). We report the effects of the partner PGI controlling for ego’s first observed scores for height, thereby directly controlling for observed phenotypic selection. For ease of comparison, we also include the social genetic effects observed for BMI, CPD, and DWP. As we can see in Figure S4 and Table S7, social genetic effects for height are insignificant in both the HRS and the ELSA, and the meta-analytic estimate combining both datasets is also insignificant. In contrast, the meta-analytic estimate for the other three phenotypes (BMI, CPD, DPW) is always significant and about 4-7 times larger in absolute size than the meta-analytic estimate for height.

Figure S4. Social genetic effects of the partner conditioning on initial behavior of ego, including height

Table S7. Random effects regression of height (t) (standardized) on the partner’s PGI conditioned on first observed height of ego in the HRS and ELSA. 1) Preferred model, 2) Model controlling for ego’s PGI, and 3) comparison model (specification as preferred model with same N as model controlling for ego’s PGI). Standard errors adjusted for clustering in individuals and households.

|  |  |  | **HRS** |  |  |  | **ELSA** |  |
| --- | --- | --- | --- | --- | --- | --- | --- | --- |
|  |  | (1) | (2) | (3) |  | (1) | (2) | (3) |
|  |  | preferred | ego PGI | comparison |  | preferred | ego PGI | comparison |
| **Height** |  |  |  |  |  |  |  |  |
| PGI height_partner_ (std.) |  | .003 | .003 | .003 |  | .002 | .001 | .001 |
|  |  | (.002) | (.002) | (.002) |  | (.003) | (.003) | (.003) |
| PGI height_ego_ (std.) |  |  | .021^***^ |  |  |  | .006^#^ |  |
|  |  |  | (.003) |  |  |  | (.003) |  |
| height_ego, t=1_ |  | .954^***^ | .947^***^ | .961^***^ |  | .976^***^ | .976^***^ | .978^***^ |
|  |  | (.007) | (.007) | (.005) |  | (.005) | (.006) | (.006) |
| height_ego, t=1_ * time |  | -.002^***^ | -.002^***^ | -.002^***^ |  | -.001^#^ | -.001^#^ | -.001^#^ |
|  |  | (.000) | (.000) | (.000) |  | (.001) | (.001) | (.001) |
| *N* observations |  | 60004 | 49102 | 49102 |  | 7551 | 6082 | 6095 |
| *N* individuals |  | 9540 | 6930 | 6930 |  | 3885 | 3022 | 3029 |
| *N* households |  | 5881 | 3478 | 3478 |  | 2460 | 1597 | 1604 |

Control variables were sex, age, age^2^ of both partners and interactions with age, age^2^, year of observation dummies, and relationship duration. PCs were the first 20 principal components.

^#^ *p* < .10, ^*^ *p* < .05, ^**^ *p* < .01, ^***^ *p* < .001

1. **Instrumental variable analyses**

We use the generalized two stage least squares (G2SLS) random-effects estimator. The partner’s polygenic index (for smoking, drinking, and BMI) acts as an instrumental variable for the partner’s actual health behavior or outcome (smoking, drinking, and BMI). For example, in the analyses on BMI, the partner’s polygenic index for BMI acts as an instrumental variable for the partner’s actual BMI. The results are presented in Table S8. Causal claims in instrumental variable analysis rely on three main assumptions: (1) the partner’s polygenic index has a causal effect on the partner’s phenotype, (2) the partner’s polygenic index only affects ego’s phenotype through the partner’s phenotype, and (3) the partner’s polygenic index and ego’s behavior do not share common causes. Our aim with these instrumental variable analyses is not to argue for strict causal evidence of the effect of partner’s behavior on ego’s behavior, but rather to examine whether the partner’s behavior is a plausible mechanism underlying the social genetic effects reported in Table S3. The outcomes (BMI, CPD, or DPW) and datasets (HRS or ELSA) in which we observe significant social genetic effects (Table S3) also show significant effects in the analyses where the partner’s phenotype is instrumented by the partner’s polygenic index (Table S8), suggesting that the partner’s phenotype is indeed a plausible mechanism for our observed social genetic effects.

Table S8. Instrumental variable random effects regression of BMI, DPW, CPD (t) (standardized) on the partner’s BMI, DPW, CPD conditioned on first observed BMI/DPW/CPD of ego in the HRS and ELSA. The partner’s polygenic index for BMI, DPW, CPD is the instrumental variable for the partner’s BMI, DPW, CPD. 1) Preferred model, 2) Model controlling for ego’s PGI, and 3) comparison model (specification as preferred model with same N as model controlling for ego’s PGI). Standard errors adjusted for clustering in individuals and households.

|  |  |  | **HRS** |  |  |  | **ELSA** |  |
| --- | --- | --- | --- | --- | --- | --- | --- | --- |
|  |  | (1) | (2) | (3) |  | (1) | (2) | (3) |
|  |  | preferred | ego PGI | comparison |  | preferred | ego PGI | comparison |
| **BMI** |  |  |  |  |  |  |  |  |
| IV BMI_partner_ (std.) |  | .050^***^ | .034^*^ | .038^*^ |  | .019 | .020 | .018 |
|  |  | (.013) | (.015) | (.015) |  | (.019) | (.021) | (.021) |
| PGI BMI_ego_ (std.) |  |  | .047^***^ |  |  |  | .040^***^ |  |
|  |  |  | (.006) |  |  |  | (.008) |  |
| BMI_ego, t=1_ |  | .869^***^ | .856^***^ | .872^***^ |  | .950^***^ | .931^***^ | .947^***^ |
|  |  | (.008) | (.010) | (.010) |  | (.013) | (.016) | (.015) |
| BMI_ego, t=1_ * time |  | -.004^***^ | -.004^***^ | -.004^***^ |  | -.007^***^ | -.007^***^ | -.007^***^ |
|  |  | (.001) | (.001) | (.001) |  | (.001) | (.002) | (.002) |
| *N* observations |  | 58222 | 47733 | 47733 |  | 11309 | 8836 | 8836 |
| *N* individuals |  | 9480 | 6890 | 6890 |  | 4266 | 3237 | 3237 |
| *N* households |  | 6239 | 3404 | 3404 |  | 3295 | 1727 | 1727 |
| **CPD** |  |  |  |  |  |  |  |  |
| IV CPD_partner_ (std.) |  | .185^*^ | .253^*^ | .251^*^ |  | .421^#^ | .362 | .354 |
|  |  | (.093) | (.118) | (.116) |  | (.227) | (.260) | (.255) |
| PGI CPD_ego_ (std.) |  |  | .007 |  |  |  | -.016 |  |
|  |  |  | (.007) |  |  |  | (.015) |  |
| CPD_ego, t=1_ |  | .895^***^ | .856^***^ | .857^***^ |  | .776^***^ | .765^***^ | .765^***^ |
|  |  | (.041) | (.055) | (.054) |  | (.069) | (.086) | (.085) |
| CPD_ego, t=1_ * time |  | -.035^***^ | -.033^***^ | -.033^***^ |  | -.031^***^ | -.031^***^ | -.031^***^ |
|  |  | (.002) | (.002) | (.002) |  | (.004) | (.004) | (.004) |
| *N* observations |  | 59275 | 48531 | 48531 |  | 25649 | 18200 | 18200 |
| *N* individuals |  | 9525 | 6919 | 6919 |  | 4942 | 3305 | 3305 |
| *N* households |  | 6242 | 3405 | 3405 |  | 3494 | 1692 | 1692 |
| **DPW** |  |  |  |  |  |  |  |  |
| IV DPW_partner_ (std.) |  | .159^**^ | .181^**^ | .184^**^ |  | .174^**^ | .135^#^ | .144^*^ |
|  |  | (.053) | (.065) | (.064) |  | (.058) | (.072) | (.071) |
| PGI DPW_ego_ (std.) |  |  | .027^***^ |  |  |  | .047^***^ |  |
|  |  |  | (.006) |  |  |  | (.009) |  |
| DPW_ego, t=1_ |  | .725^***^ | .726^***^ | .728^***^ |  | .588^***^ | .592^***^ | .598^***^ |
|  |  | (.024) | (.031) | (.031) |  | (.025) | (.032) | (.031) |
| DPW_ego, t=1_ * time |  | -.009^***^ | -.008^***^ | -.008^***^ |  | -.001 | -.000 | -.000 |
|  |  | (.001) | (.001) | (.001) |  | (.001) | (.001) | (.001) |
| *N* observations |  | 51432 | 42899 | 42899 |  | 23128 | 16693 | 16693 |
| *N* individuals |  | 9121 | 6855 | 6855 |  | 4888 | 3342 | 3342 |
| *N* households |  | 5987 | 3420 | 3420 |  | 3478 | 1725 | 1725 |
| controls |  | Yes | Yes | Yes |  | Yes | Yes | Yes |
| wave dummies |  | Yes | Yes | Yes |  | Yes | Yes | Yes |
| PCs_ego_ |  | No | Yes | No |  | No | Yes | No |
| PCs_partner_ |  | Yes | Yes | Yes |  | Yes | Yes | Yes |

Control variables were sex, age, age^2^ of both partners and interactions with age, age^2^, year of observation dummies, and relationship duration. PCs were the first 20 principal components.

^#^ *p* < .10, ^*^ *p* < .05, ^**^ *p* < .01, ^***^ *p* < .001

1. **Analyses using change scores**

Table S9. Random effects regression of change in BMI, DPW, CPD between the current wave and first observed wave (t) (standardized) on the partner’s PGI in the HRS and ELSA. 1) Preferred model, 2) Model controlling for ego’s PGI, and 3) comparison model (specification as preferred model with same N as model controlling for ego’s PGI). Standard errors adjusted for clustering in individuals and households.

|  |  |  | **HRS** |  |  |  | **ELSA** |  |
| --- | --- | --- | --- | --- | --- | --- | --- | --- |
|  |  | (1) | (2) | (3) |  | (1) | (2) | (3) |
|  |  | preferred | ego PGI | comparison |  | preferred | ego PGI | comparison |
| **Change in BMI** |  |  |  |  |  |  |  |  |
| PGI BMI_partner_ (std.) |  | .031^***^ | .021^*^ | .024^*^ |  | .015 | .014 | .012 |
|  |  | (.009) | (.010) | (.010) |  | (.013) | (.014) | (.014) |
| PGI BMI_ego_ (std.) |  |  | .087^***^ |  |  |  | .080^***^ |  |
|  |  |  | (.011) |  |  |  | (.016) |  |
| BMI_ego, t=1_ |  | -.105^***^ | -.133^***^ | -.102^***^ |  | .013 | -.021 | .010 |
|  |  | (.015) | (.018) | (.018) |  | (.024) | (.030) | (.028) |
| BMI_ego, t=1_ * time |  | -.008^***^ | -.007^***^ | -.007^***^ |  | -.014^***^ | -.013^***^ | -.012^***^ |
|  |  | (.001) | (.002) | (.002) |  | (.003) | (.003) | (.003) |
| *N* observations |  | 59325 | 48572 | 48572 |  | 11728 | 9155 | 9155 |
| *N* individuals |  | 9522 | 6914 | 6914 |  | 4311 | 3262 | 3262 |
| *N* households |  | 5879 | 3477 | 3477 |  | 2729 | 1680 | 1680 |
| **Change in CPD** |  |  |  |  |  |  |  |  |
| PGI CPD_partner_ (std.) |  | .011^#^ | .014^*^ | .014^*^ |  | .016^*^ | .014 | .014 |
|  |  | (.006) | (.007) | (.007) |  | (.008) | (.009) | (.009) |
| PGI CPD_ego_ (std.) |  |  | .013^*^ |  |  |  | -.007 |  |
|  |  |  | (.006) |  |  |  | (.010) |  |
| CPD_ego, t=1_ |  | -.321^***^ | -.326^***^ | -.325^***^ |  | -.243^***^ | -.266^***^ | -.267^***^ |
|  |  | (.023) | (.024) | (.024) |  | (.044) | (.061) | (.061) |
| CPD_ego, t=1_ * time |  | -.039^***^ | -.038^***^ | -.038^***^ |  | -.037^***^ | -.036^***^ | -.036^***^ |
|  |  | (.002) | (.002) | (.002) |  | (.004) | (.005) | (.005) |
| *N* observations |  | 60029 | 49108 | 49108 |  | 25740 | 18270 | 18270 |
| *N* individuals |  | 9546 | 6931 | 6931 |  | 4943 | 3305 | 3305 |
| *N* households |  | 5885 | 3478 | 3478 |  | 3311 | 1673 | 1673 |
| **Change in DPW** |  |  |  |  |  |  |  |  |
| PGI DPW_partner_ (std.) |  | .021^**^ | .023^**^ | .023^**^ |  | .029^**^ | .021^#^ | .023^*^ |
|  |  | (.008) | (.009) | (.009) |  | (.010) | (.012) | (.012) |
| PGI DPW_ego_ (std.) |  |  | .039^***^ |  |  |  | .058^***^ |  |
|  |  |  | (.009) |  |  |  | (.011) |  |
| DPW_ego, t=1_ |  | -.283^***^ | -.264^***^ | -.258^***^ |  | -.430^***^ | -.440^***^ | -.428^***^ |
|  |  | (.012) | (.013) | (.013) |  | (.013) | (.016) | (.015) |
| DPW_ego, t=1_ * time |  | -.013^***^ | -.013^***^ | -.013^***^ |  | -.001 | -.001 | -.001 |
|  |  | (.001) | (.001) | (.001) |  | (.001) | (.002) | (.002) |
| *N* observations |  | 52023 | 43358 | 43358 |  | 24179 | 17485 | 17485 |
| *N* individuals |  | 9140 | 6866 | 6866 |  | 4911 | 3350 | 3350 |
| *N* households |  | 5584 | 3462 | 3462 |  | 3264 | 1703 | 1703 |
| controls |  | Yes | Yes | Yes |  | Yes | Yes | Yes |
| wave dummies |  | Yes | Yes | Yes |  | Yes | Yes | Yes |
| PCs_ego_ |  | No | Yes | No |  | No | Yes | No |
| PCs_partner_ |  | Yes | Yes | Yes |  | Yes | Yes | Yes |

Control variables were sex, age, age^2^ of both partners and interactions with age, age^2^, year of observation dummies, and relationship duration. PCs were the first 20 principal components.

^#^ *p* < .10, ^*^ *p* < .05, ^**^ *p* < .01, ^***^ *p* < .001

1. **Social genetic effects by years between current and baseline observation**

Table S10. Random effects regression of BMI, DPW, CPD (t) (standardized) on the partner’s PGI conditioned on first observed BMI/DPW/CPD of ego in the HRS and ELSA, including the interaction between partner’s PGI and the number of years since ego’s first observed BMI/DPW/CPD. 1) Preferred model, 2) Model controlling for ego’s PGI, and 3) comparison model (specification as preferred model with same N as model controlling for ego’s PGI). Standard errors adjusted for clustering in individuals and households.

|  |  |  | **HRS** |  |  |  | **ELSA** |  |
| --- | --- | --- | --- | --- | --- | --- | --- | --- |
|  |  | (1) | (2) | (3) |  | (1) | (2) | (3) |
|  |  | preferred | ego PGI | comparison |  | preferred | ego PGI | comparison |
| **BMI** |  |  |  |  |  |  |  |  |
| PGI BMI_partner_ (std.) |  | .010^*^ | .007 | .006 |  | .007 | .000 | .001 |
|  |  | (.005) | (.006) | (.006) |  | (.008) | (.009) | (.009) |
| PGI BMI_ego_ (std.) |  |  |  | .046^***^ |  |  |  | .041^***^ |
|  |  |  |  | (.006) |  |  |  | (.008) |
| BMI_ego, t=1_ |  | .878^***^ | .879^***^ | .863^***^ |  | .952^***^ | .951^***^ | .935^***^ |
|  |  | (.008) | (.009) | (.010) |  | (.012) | (.014) | (.015) |
| BMI_ego, t=1_ * time |  | -.004^***^ | -.004^***^ | -.004^***^ |  | -.007^***^ | -.006^***^ | -.006^***^ |
|  |  | (.001) | (.001) | (.001) |  | (.001) | (.002) | (.002) |
| PGI BMI_partner_ * time |  | .001 | .001 | .001 |  | .000 | .001 | .001 |
|  |  | (.001) | (.001) | (.001) |  | (.001) | (.001) | (.001) |
| *N* observations |  | 59325 | 48572 | 48572 |  | 11728 | 9155 | 9155 |
| *N* individuals |  | 9522 | 6914 | 6914 |  | 4311 | 3262 | 3262 |
| *N* households |  | 5879 | 3477 | 3477 |  | 2729 | 1680 | 1680 |
| **CPD** |  |  |  |  |  |  |  |  |
| PGI CPD_partner_ (std.) |  | .013^#^ | .018^*^ | .018^*^ |  | .012 | .011 | .011 |
|  |  | (.007) | (.008) | (.008) |  | (.011) | (.013) | (.013) |
| PGI CPD_ego_ (std.) |  |  |  | .012^*^ |  |  |  | -.006 |
|  |  |  |  | (.006) |  |  |  | (.010) |
| CPD_ego, t=1_ |  | .961^***^ | .957^***^ | .956^***^ |  | .887^***^ | .864^***^ | .865^***^ |
|  |  | (.022) | (.023) | (.023) |  | (.041) | (.057) | (.057) |
| CPD_ego, t=1_ * time |  | -.037^***^ | -.036^***^ | -.036^***^ |  | -.034^***^ | -.034^***^ | -.034^***^ |
|  |  | (.002) | (.002) | (.002) |  | (.003) | (.004) | (.004) |
| PGI CPD_partner_ * time |  | -.000 | -.001 | -.001 |  | .000 | .000 | .000 |
|  |  | (.001) | (.001) | (.001) |  | (.001) | (.001) | (.001) |
| *N* observations |  | 60029 | 49108 | 49108 |  | 25740 | 18270 | 18270 |
| *N* individuals |  | 9546 | 6931 | 6931 |  | 4943 | 3305 | 3305 |
| *N* households |  | 5885 | 3478 | 3478 |  | 3311 | 1673 | 1673 |
| **DPW** |  |  |  |  |  |  |  |  |
| PGI DPW_partner_ (std.) |  | .011^#^ | .015^*^ | .015^*^ |  | .026^**^ | .025^*^ | .023^*^ |
|  |  | (.006) | (.007) | (.007) |  | (.010) | (.012) | (.012) |
| PGI DPW_ego_ (std.) |  |  |  | .029^***^ |  |  |  | .047^***^ |
|  |  |  |  | (.006) |  |  |  | (.009) |
| DPW_ego, t=1_ |  | .793^***^ | .811^***^ | .806^***^ |  | .661^***^ | .662^***^ | .653^***^ |
|  |  | (.009) | (.010) | (.010) |  | (.010) | (.012) | (.013) |
| DPW_ego, t=1_ * time |  | -.010^***^ | -.009^***^ | -.009^***^ |  | -.001 | -.001 | -.001 |
|  |  | (.001) | (.001) | (.001) |  | (.001) | (.001) | (.001) |
| PGI DPW_partner_ * time |  | .001 | .000 | .000 |  | -.000 | -.001 | -.001 |
|  |  | (.001) | (.001) | (.001) |  | (.001) | (.001) | (.001) |
| *N* observations |  | 52023 | 43358 | 43358 |  | 24179 | 17485 | 17485 |
| *N* individuals |  | 9140 | 6866 | 6866 |  | 4911 | 3350 | 3350 |
| *N* households |  | 5584 | 3462 | 3462 |  | 3264 | 1703 | 1703 |
| controls |  | Yes | Yes | Yes |  | Yes | Yes | Yes |
| wave dummies |  | Yes | Yes | Yes |  | Yes | Yes | Yes |
| PCs_ego_ |  | No | Yes | No |  | No | Yes | No |
| PCs_partner_ |  | Yes | Yes | Yes |  | Yes | Yes | Yes |

Control variables were sex, age, age^2^ of both partners and interactions with age, age^2^, year of observation dummies, and relationship duration. PCs were the first 20 principal components.

^#^ *p* < .10, ^*^ *p* < .05, ^**^ *p* < .01, ^***^ *p* < .001

1. **Analyses controlling for baseline difference in behavior between ego and partner**

Table S11. Random effects regression of BMI, DPW, CPD (t) (standardized) on the partner’s PGI conditioned on first observed BMI/DPW/CPD of ego in the HRS and ELSA, controlling for baseline difference in behavior between ego and partner. 1) Preferred model, 2) Model controlling for ego’s PGI, and 3) comparison model (specification as preferred model with same N as model controlling for ego’s PGI). Standard errors adjusted for clustering in individuals and households.

|  |  |  | **HRS** |  |  |  | **ELSA** |  |
| --- | --- | --- | --- | --- | --- | --- | --- | --- |
|  |  | (1) | (2) | (3) |  | (1) | (2) | (3) |
|  |  | preferred | ego PGI | comparison |  | preferred | ego PGI | comparison |
| **BMI** |  |  |  |  |  |  |  |  |
| PGI BMI_partner_ (std.) |  | .017^***^ | .012^*^ | .014^*^ |  | .007 | .008 | .007 |
|  |  | (.005) | (.005) | (.005) |  | (.007) | (.007) | (.007) |
| PGI BMI_ego_ (std.) |  |  | .046^***^ |  |  |  | .041^***^ |  |
|  |  |  | (.006) |  |  |  | (.008) |  |
| BMI dif._ego - partner, t=1_ |  | -.016^**^ | -.018^**^ | -.019^**^ |  | -.006 | -.011 | -.011 |
|  |  | (.005) | (.007) | (.007) |  | (.008) | (.008) | (.008) |
| BMI_ego, t=1_ |  | .882^***^ | .868^***^ | .884^***^ |  | .953^***^ | .937^***^ | .953^***^ |
|  |  | (.008) | (.010) | (.009) |  | (.012) | (.015) | (.014) |
| BMI_ego, t=1_ * time |  | -.004^***^ | -.004^***^ | -.004^***^ |  | -.007^***^ | -.006^***^ | -.006^***^ |
|  |  | (.001) | (.001) | (.001) |  | (.001) | (.002) | (.002) |
| *N* observations |  | 59306 | 48556 | 48556 |  | 11705 | 9139 | 9139 |
| *N* individuals |  | 9517 | 6912 | 6912 |  | 4300 | 3254 | 3254 |
| *N* households |  | 5874 | 3475 | 3475 |  | 2718 | 1672 | 1672 |
| **CPD** |  |  |  |  |  |  |  |  |
| PGI CPD_partner_ (std.) |  | .011^#^ | .014^*^ | .014^*^ |  | .016^*^ | .015^#^ | .015^#^ |
|  |  | (.006) | (.007) | (.006) |  | (.007) | (.009) | (.009) |
| PGI CPD_ego_ (std.) |  |  | .012^*^ |  |  |  | -.005 |  |
|  |  |  | (.006) |  |  |  | (.010) |  |
| CPD dif._ego - partner, t=1_ |  | -.015 | -.005 | -.005 |  | -.029 | -.053^*^ | -.052^*^ |
|  |  | (.010) | (.011) | (.011) |  | (.018) | (.023) | (.023) |
| CPD_ego, t=1_ |  | .970^***^ | .960^***^ | .960^***^ |  | .904^***^ | .895^***^ | .893^***^ |
|  |  | (.022) | (.024) | (.024) |  | (.040) | (.056) | (.056) |
| CPD_ego, t=1_ * time |  | -.037^***^ | -.036^***^ | -.036^***^ |  | -.034^***^ | -.034^***^ | -.034^***^ |
|  |  | (.002) | (.002) | (.002) |  | (.003) | (.004) | (.004) |
| *N* observations |  | 60028 | 49107 | 49107 |  | 25740 | 18270 | 18270 |
| *N* individuals |  | 9545 | 6930 | 6930 |  | 4943 | 3305 | 3305 |
| *N* households |  | 5885 | 3478 | 3478 |  | 3311 | 1673 | 1673 |
| **DPW** |  |  |  |  |  |  |  |  |
| PGI DPW_partner_ (std.) |  | .016^**^ | .017^**^ | .017^**^ |  | .024^**^ | .019^*^ | .020^*^ |
|  |  | (.005) | (.006) | (.006) |  | (.008) | (.009) | (.009) |
| PGI DPW_ego_ (std.) |  |  | .029^***^ |  |  |  | .049^***^ |  |
|  |  |  | (.006) |  |  |  | (.009) |  |
| DPW dif._ego - partner, t=1_ |  | -.011 | -.008 | -.008 |  | -.082^***^ | -.083^***^ | -.081^***^ |
|  |  | (.007) | (.009) | (.009) |  | (.008) | (.010) | (.010) |
| DPW_ego, t=1_ |  | .797^***^ | .809^***^ | .814^***^ |  | .661^***^ | .654^***^ | .664^***^ |
|  |  | (.009) | (.010) | (.010) |  | (.010) | (.013) | (.012) |
| DPW_ego, t=1_ * time |  | -.009^***^ | -.009^***^ | -.009^***^ |  | -.001 | -.001 | -.001 |
|  |  | (.001) | (.001) | (.001) |  | (.001) | (.001) | (.001) |
| *N* observations |  | 52012 | 43347 | 43347 |  | 24176 | 17483 | 17483 |
| *N* individuals |  | 9139 | 6865 | 6865 |  | 4909 | 3349 | 3349 |
| *N* households |  | 5583 | 3461 | 3461 |  | 3262 | 1702 | 1702 |
| controls |  | Yes | Yes | Yes |  | Yes | Yes | Yes |
| wave dummies |  | Yes | Yes | Yes |  | Yes | Yes | Yes |
| PCs_ego_ |  | No | Yes | No |  | No | Yes | No |
| PCs_partner_ |  | Yes | Yes | Yes |  | Yes | Yes | Yes |

Control variables were sex, age, age^2^ of both partners and interactions with age, age^2^, year of observation dummies, and relationship duration. PCs were the first 20 principal components.

^#^ *p* < .10, ^*^ *p* < .05, ^**^ *p* < .01, ^***^ *p* < .001

1. **Multilevel multivariate analyses**

Multilevel multivariate analyses introduce an additional level for the outcome variables, such that multiple outcome variables can be included within one model. In our case, this allows us to analyze our three phenotypes (BMI, DPW, and CPD) together. We add the phenotypes as dummy variables, with BMI as the reference category. We run the same sets of models as in Table S3, but this time combining the data from the three different phenotypes. This means that the total *N* observations is the sum of the *N* observations of the separate analyses per phenotype (see Table S3). In the preferred model (model 1), we find social genetic effects both in the HRS and the ELSA. In the models where we require both partners to have participated in the genetic part of the study (models 2 and 3), effects are reduced in the ELSA. However, adding the ego PGI does not reduce the social genetic effect (compare models 2 and 3). In the main text, we report meta-analytic estimates per phenotype (BMI, DPW, and CPD) that aggregate estimates from the HRS and ELSA. The meta-analytic estimates show significant social genetic effects for each phenotype also when both partners participated in the genetic part of the study.

Table S12. Random effects multivariate regression of phenotype (t) (standardized) on the partner’s PGI conditioned on first observed phenotype of ego in the HRS and ELSA.
1) Preferred model, 2) Model controlling for ego’s PGI, and 3) comparison model (specification as preferred model with same N as model controlling for ego’s PGI). Standard errors adjusted for clustering in individuals and households.

|  |  |  | **HRS** |  |  |  | **ELSA** |  |
| --- | --- | --- | --- | --- | --- | --- | --- | --- |
|  |  | (1) | (2) | (3) |  | (1) | (2) | (3) |
|  |  | preferred | ego PGI | comparison |  | preferred | ego PGI | comparison |
| PGI_partner_ (std.) |  | .021^***^ | .020^***^ | .021^***^ |  | .014^**^ | .011^#^ | .011^#^ |
|  |  | (.004) | (.004) | (.004) |  | (.005) | (.006) | (.006) |
| PGI_ego_ (std.) |  |  | .044^***^ |  |  |  | .042^***^ |  |
|  |  |  | (.004) |  |  |  | (.006) |  |
| phenotype_ego, t=1_ |  | .906^***^ | .905^***^ | .913^***^ |  | .796^***^ | .785^***^ | .792^***^ |
|  |  | (.009) | (.010) | (.010) |  | (.019) | (.026) | (.026) |
| phenotype_ego, t=1_ * time |  | -.018^***^ | -.018^***^ | -.018^***^ |  | -.012^***^ | -.013^***^ | -.013^***^ |
|  |  | (.001) | (.001) | (.001) |  | (.002) | (.002) | (.002) |
| Outcome (DPW) |  | -.018 | -.015 | -.015 |  | -.070^***^ | -.055^**^ | -.055^**^ |
|  |  | (.009) | (.011) | (.011) |  | (.014) | (.017) | (.017) |
| Outcome (CPD) |  | -.041^***^ | -.058^***^ | -.058^***^ |  | -.051^***^ | -.045^**^ | -.045^**^ |
|  |  | (.009) | (.010) | (.010) |  | (.013) | (.016) | (.016) |
| *N* observations |  | 171377 | 141038 | 141038 |  | 61647 | 44910 | 44910 |
| *N* individuals |  | 9546 | 6931 | 6931 |  | 5016 | 3387 | 3387 |
| *N* households |  | 5885 | 3478 | 3478 |  | 3344 | 1715 | 1715 |
| controls |  | Yes | Yes | Yes |  | Yes | Yes | Yes |
| wave dummies |  | Yes | Yes | Yes |  | Yes | Yes | Yes |
| PCs_ego_ |  | No | Yes | No |  | No | Yes | No |
| PCs_partner_ |  | Yes | Yes | Yes |  | Yes | Yes | Yes |

Control variables were phenotype, sex, age, age^2^ of both partners and interactions with age, age^2^, year of observation dummies, and relationship duration.

^#^ *p* < .10, ^*^ *p* < .05, ^**^ *p* < .01, ^***^ *p* < .001

**Supplementary References**

Ajnakina O, Steptoe A (2019) The English Longitudinal Study of Aging (ELSA). Polygenic scores

Becker J, Burik CAP, Goldman G, et al (2021) Resource profile and user guide of the Polygenic Index Repository. Nat Hum Behav 5:. https://doi.org/10.1038/s41562-021-01119-3

Liu M, Jiang Y, Wedow R, et al (2019) Association studies of up to 1.2 million individuals yield new insights into the genetic etiology of tobacco and alcohol use. Nat Genet 51:237–244. https://doi.org/10.1038/s41588-018-0307-5

Locke AE, Kahali B, Berndt SI, et al (2015) Genetic studies of body mass index yield new insights for obesity biology. Nature 518:197–206. https://doi.org/10.1038/nature14177

Ware E, Gard A, Schmitz L, Faul J (2021) HRS Documentation Report: HRS Polygenic Scores – Release 4.3, 2006-2012 Genetic Data

Wood AR, Esko T, Yang J, et al (2014) Defining the role of common variation in the genomic and biological architecture of adult human height. Nat Genet 46:1173–1186. https://doi.org/10.1038/ng.3097

Yengo L, Sidorenko J, Kemper KE, et al (2018) Meta-analysis of genome-wide association studies for height and body mass index in ~700 000 individuals of European ancestry. Hum Mol Genet 27:3641–3649. https://doi.org/10.1093/hmg/ddy271
